# When a negative (charge) is not a positive: sialylation and its role in cancer mechanics and progression

**DOI:** 10.3389/fonc.2024.1487306

**Published:** 2024-11-19

**Authors:** Issa Funsho Habeeb, Toheeb Eniola Alao, Daniella Delgado, Alexander Buffone

**Affiliations:** ^1^ Department of Biomedical Engineering, New Jersey Institute of Technlogy, Newark, NJ, United States; ^2^ Chemical and Materials Engineering, New Jersey Institute of Technlogy, Newark, NJ, United States

**Keywords:** sialylation, cancer, glycocalyx, mechanobiology, metastasis, migration

## Abstract

Sialic acids and sialoglycans are critical actors in cancer progression and metastasis. These terminal sugar residues on glycoproteins and glycolipids modulate key cellular processes such as immune evasion, cell adhesion, and migration. Aberrant sialylation is driven by overexpression of sialyltransferases, resulting in hypersialylation on cancer cell surfaces as well as enhancing tumor aggressiveness. Sialylated glycans alter the structure of the glycocalyx, a protective barrier that fosters cancer cell detachment, migration, and invasion. This bulky glycocalyx also increases membrane tension, promoting integrin clustering and downstream signaling pathways that drive cell proliferation and metastasis. They play a critical role in immune evasion by binding to Siglecs, inhibitory receptors on immune cells, which transmit signals that protect cancer cells from immune-mediated destruction. Targeting sialylation pathways presents a promising therapeutic opportunity to understand the complex roles of sialic acids and sialoglycans in cancer mechanics and progression, which is crucial for developing novel diagnostic and therapeutic strategies that can disrupt these processes and improve cancer treatment outcomes.

## Introduction

Sialylation, the enzymatic addition of sialic acid to glycoproteins and glycolipids, is a key post-translational modification that plays a significant role in cancer biology. The sialic acid moiety, often found at the terminal position of glycan chains on cell surfaces, influences a wide range of biological processes, including but not limited to cell signaling (1–5), proliferation (6–9), immune responses (10), and cellular interactions (11–14). Aberrant sialylation has been recognized as a hallmark of cancer (15), contributing to tumor progression, immune evasion (16), and metastasis (17).

The molecular basis of sialylation involves a family of enzymes known as sialyltransferases, which catalyze the transfer of sialic acid to glycan structures. These enzymes are responsible for catalyzing different linkages, such as α2,3-, α2,6-, and α2,8-sialylation, each playing distinct roles in cellular behavior (18–26). In cancer, dysregulated sialylation often results in hypersalivation, which can mediate various oncogenic processes. For instance, the upregulation of sialyltransferases ST6GAL1 has been linked to increased cancer cell aggressiveness, enhanced survival, and resistance to apoptosis in cancers such as breast, colon, prostate, and brain cancers (18, 23, 24, 27, 28).

New evidence of where sialylation occurs outside the cell membrane has also emerged as an important factor in cancer. This form of sialylation contributes to cancer cell proliferation and metastasis by altering the glycocalyx, a dense layer of glycoproteins and glycolipids on the cell surface (29, 30). A sialylated glycocalyx not only provides a protective barrier against the immune system but also mediates cancer cell detachment and migration (31) by increasing mechanical tension at the cell membrane (32). The mechanical properties of the glycocalyx, such as its stiffness and bulk, influence membrane dynamics and signal transduction, promoting cancer cell invasion and metastatic potential (33–35).

Sialylation also plays a critical role in immune evasion, with sialic acid residues acting as ligands for Siglecs, a family of immune inhibitory receptors. By engaging Siglecs on immune cells, cancer cells can transmit inhibitory signals that prevent immune-mediated destruction (16, 36). For example, hypersialylated cancer cells expressing CD24 can bind to Siglec-10 on macrophages, effectively creating a “don’t eat me” signal that prevents phagocytosis (10, 16, 37–40). This immune evasion mechanism underscores the potential of targeting sialylation pathways as a therapeutic strategy in cancer treatment. Given its widespread impact on cancer biology, sialylation serves as a promising biomarker for cancer diagnosis and prognosis. Aberrant sialylation patterns have been associated with more aggressive tumor phenotypes and poor patient outcomes (27, 41–43), making it a valuable target for early detection and therapeutic intervention (44–46). Overall, this review aims to highlight the multifaceted roles of sialylation in cancer, emphasizing its contribution to tumor progression, immune evasion, and potential as a therapeutic target.

## The molecular basis of sialylation

Sialylation is a well-regulated biological process that involves the addition of negatively charged sialic acid sugar residues to the glycoproteins and glycolipids at the terminal position of the glycan (N-, O-linked glycan or glycolipids) to mediate protein stability, cell–cell communication, and immune response (47). Sialic acids are a family of α-keto acids with unique structural features, containing nine-carbon backbone sugar molecules with an amino group at position 5 and a carboxyl group at position 1 (48). More than 50 sialic acid isoforms have been found in nature, with the most abundant being N-acetylneuraminic acid (Neu5Ac) (49, 50). N-acetylneuraminic acid (Neu5Ac) is derived from cytidine monophosphate sugar nucleotide CMP-Neu5Ac and can undergo acetylation, methylation, and other modifications. Sialylation is mediated by sialyltransferases that catalyze the transfer of sialic acid from the charged donor molecule, cytidine monophosphate–sialic acid (CMP–sialic acid), to the growing glycan chains in either an α2,3, α2,6, or α2,8 linkage depending on the activity of the sialyltransferases and the substrate glycan (48, 51).

**Figure 1:**
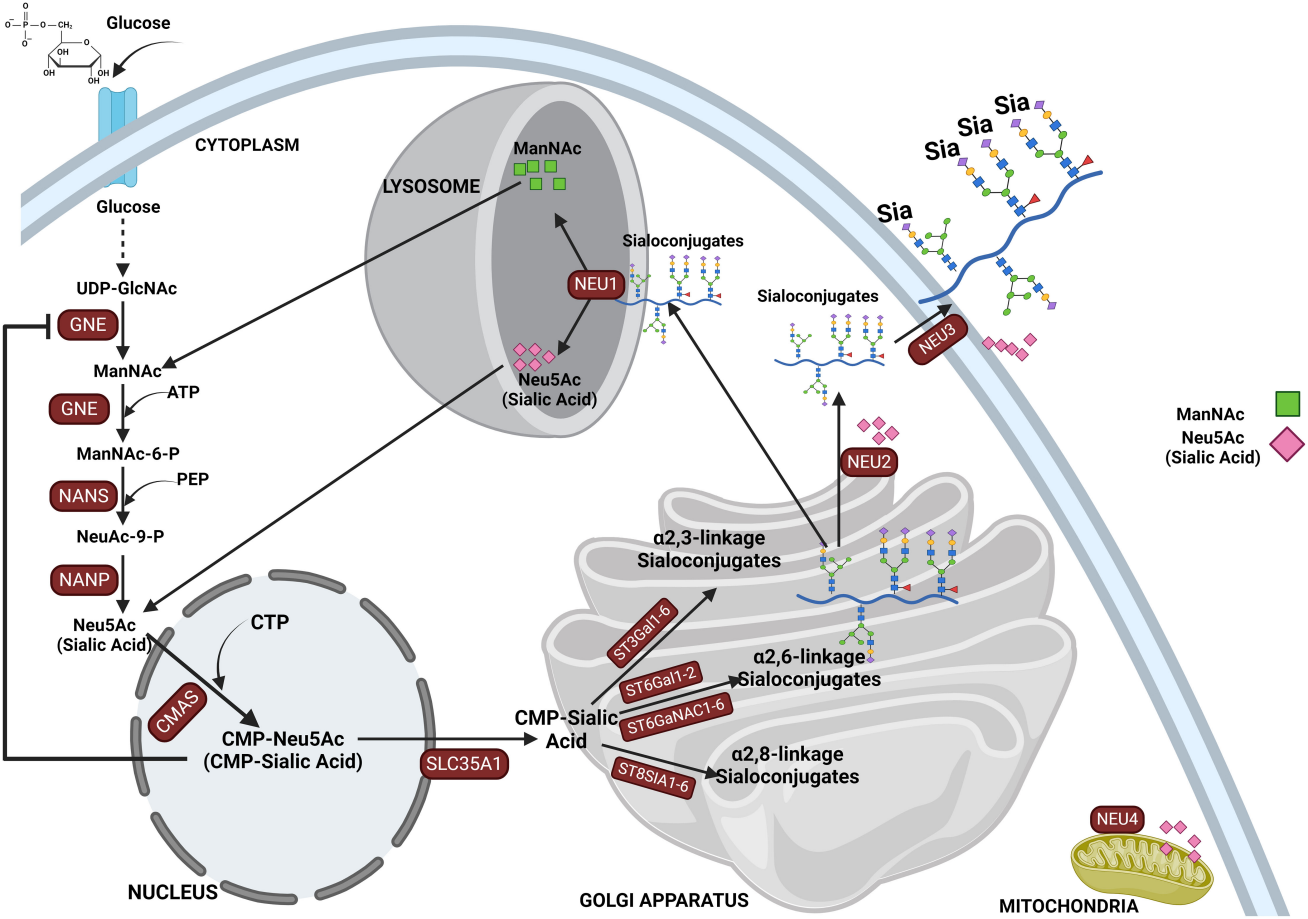
This figure depicts the sialylation pathway, illustrating the sequential biochemical steps involved in the synthesis and recycling of sialic acids. The pathway begins with the nucleotide sugar UDP-GlcNAc, produced via the hexosamine pathway, which is converted to ManNAc by UDP-GlcNAc 2-epimerase (GNE). ManNAc serves as the metabolic precursor for sialic acid synthesis, leading to the production of Neu5Ac in the cytosol. Neu5Ac then enters the nucleus, where it is converted into CMP-Neu5Ac. CMP-Neu5Ac is subsequently transported into the Golgi apparatus, where it is utilized by various sialyltransferases (ST3GAL1-6, ST6GAL1-2/ST6GALNAC1-6, and ST8SIA4) to synthesize α-2,3-, α-2,6-, and α-2,8-linked sialoglycoproteins or gangliosides. The final step in the pathway involves the recycling of sialosides by neuraminidases, which break them down into sialic acid monomers for reuse. This comprehensive depiction highlights the critical processes of sialic acid biosynthesis, modification, and recycling in cellular glycosylation.

Sialic acid biosynthesis progresses through a four-step process involving three different enzymes. GNE is the bifunctional enzyme that catalyzes the initial two steps in the biosynthesis pathway (**Figure 1**). This pathway begins in the cytosol with the formation of N-acetylmannosamine (ManNAc) from UDP-GlcNAc using the epimerase function of the GNE, followed by the subsequent phosphorylation of ManNAc to N-acetylmannosamine-6-phosphate (ManNAc-6-P) and conversion to N-acetylneuraminic acid-9-phosphate (Neu5Ac-9-P). Dephosphorylation of Neu5Ac-9-P produces free Neu5Ac, which is then transported to the nucleus for subsequent activation into CMP-Neu5Ac by CMP–sialic acid synthetase (CMAS) (51). After transport to the Golgi, the active CMP-Neu5Ac donors are used by sialyltransferases to catalyze the addition of sialic acid to the glycoconjugates. Subsequent secretion of these sialylated glycans to the cell surface significantly contributes to the biophysical properties of cells through the negative charge carried by the sialic acid (52).

Post-glycosylation modifications in α-linked sialoglycoconjugates are common in mammalian cells, with O-acetylation of N-acetylneuraminic acid (Neu5Ac) being the most frequent. O-acetylation is an important modification, occurring at positions C4, C7, C8, and C9 on N-acetylneuraminic acid (Neu5Ac) (53, 54). More frequently, they occur at positions C7, C8, and C9 across various species. However, it is also possible for O-acetylation to take place at the C4 position, which is directly connected to the pyranosyl ring, though this form is less common. These modifications influence the stability, recognition, and function of glycoproteins and glycolipids, impacting cellular interactions via immune modulation, pathogen binding, and overall cell signaling processes in various biological systems (55). In mammals, acetylation of sialic acid occurs at position C7 or C9 and is mediated by the enzyme CASD1, which utilizes acetyl-CoA as the acetyl donor. Following acetylation, sialyltransferases transfer O-acetylated sialic acids onto glycoproteins and glycolipids, which are then processed through the secretory pathway. Notably, some O-acetylated glycoproteins and glycolipids carrying sialic acids may remain within the Golgi for reasons not yet defined. However, deacetylation is carried out by the sialic acid esterase (SIAE), which is found both inside and outside of cells (54).

There are several reports of an increased O-acetylated sialic acid in cancers, which are implicated in tumor progression by inhibiting immune surveillance and enhancing oncogenic signaling. An O-acetylated sialyl-Tn has been reported to be involved in ovarian cancer-associated antigenicity. It was shown that modification of Sia with O-acetyl groups was critical for the recognition (56). 9-O-Ac-GD3, a modified ganglioside, is also found in several other cancers, including neuroblastoma (57), acute lymphoblastic leukemia (ALL) (58, 59), medulloblastoma (60), and breast cancer (61), but are typically rare in healthy adult tissues. Furthermore, sialic acids exist with different other modifications in which the hydroxyl groups may either be methylated or esterified with acetyl, lactyl, phosphate, or sulfate groups (62). It is critical to note that methylation of sialic acids plays a crucial role in cancer progression including signaling and cancer migration. Methylated sialic acid affects cancer cell adhesion and detachment mechanisms that are critical for metastasis. Specifically, they contribute to the mechanical stress and electrostatic forces that influence cancer cell migration. Recent findings highlight the association between the elevated production of sialic acids and increased methylation in cancer cells. This methylation alters the sialic acid’s role in cell-surface interactions, facilitating cancer cells’ ability to evade immune detection and promoting metastatic behavior (63, 64).

Several sugars make up both N-, O-linked, and glycolipid glycans, among which N-acetylneuraminic acid (Neu5Ac) is the most common sialic acid in humans (65–67) and holds great importance since they are termed “terminal” or capping sugars. Sialyltransferases catalyze the bond between sialic acid and glycan receptors, mediating the glycosidic bond formation between the sialic acid donor at Carbon-2 and the glycan receptor at Carbon-3, Carbon-6, or Carbon-8 hydroxyl positions, and are thus named ST3, ST6, or ST8, respectively, depending on the carbon number of the glycan onto which sialic acid is added (48, 65, 68) (see **Table 1**). The level of 2,6 sialylation is an important marker of cancer progression as α2,6-galactoside sialyltransferase 1 (ST6Gal1) (90) upregulation has been linked to aggressiveness of cancer cells (18, 27, 33, 44, 91–93) including colorectal (92, 94), gastric carcinoma (95), lungs (96), and brain (18). Additionally, another 2,6 siayltransferase, ST6GalNAc1, has been reported to regulate cancer cell adhesion and invasion in prostate cancer (46, 91).

**Table 1:** Sialyltransferases and related genes.

Gene name	Protein name	Function	Expressed in	Reference
GNE/Gne	Glucosamine (UDP-N-acetyl)-2-epimerase	Bifunctional enzyme that initiates and regulates the biosynthesis of N-acetylneuraminic acid (NeuAc). Catalyzes the conversion of UDP-GlcNAc to ManNAc	Liver and colon	(69)
GNE/Gne	N-acetylmannosamine kinase	Catalyzes the conversion of ManNAc to ManNAc-6P	Liver and colon	(69)
NANS/Nans	N-acetylneuraminate synthase	Catalyzes the formation of NeuAC-9-P from ManNAc-6P	Colon and prostate	(70)
NANP/Nanp	N-acetylneuraminic acid phosphatase	Involved in N-acetylneuraminate biosynthetic process. Convert Neu5Ac	Adrenal, testis, kidney	(71, 72)
CMAS/Cmas	cytidine monophosphate N-acetylneuraminic acid synthetase	Encodes an enzyme that converts N-acetylneuraminic acid (NeuNAc) to cytidine 5’-monophosphate N-acetylneuraminic acid (CMP-NeuNAc)	Testis and colon	(73)
ST3GAL1/St3gal1	ST3 beta-galactoside alpha-2,3-sialyltransferase 1	Catalyzes the transfer of sialic acid from CMP–sialic acid to galactose-containing substrates in an alpha 2,3 linkage	Thyroid, kidney, and heart	(74)
ST3GAL2/St3gal2	ST3 beta-galactoside alpha-2,3-sialyltransferase 2		Appendix and spleen	(75)
ST3GAL3/St3gal3	ST3 beta-galactoside alpha-2,3-sialyltransferase 3		Testis and fat	(76, 77)
ST3GAL4/St3gal4	ST3 beta-galactoside alpha-2,3-sialyltransferase 4		Adrenal and ovary	(78)
ST3GAL5/St3gal5	ST3 beta-galactoside alpha-2,3-sialyltransferase 5		Liver, ovary adult and adrenal adult	(79)
ST3GAL6/St3gal6	ST3 beta-galactoside alpha-2,3-sialyltransferase 6			(21, 22)
ST6GAL1/St6gal1	ST6 beta-galactoside alpha-2,6-sialyltransferase 1	Catalyzes the transfer of sialic acid from CMP–sialic acid to galactose-containing substrates in an alpha 2,6 linkage	Liver and lymph node	(80)
ST6GAL2/St6gal2	ST6 beta-galactoside alpha-2,6-sialyltransferase 2	Catalyzes the transfer of sialic acid from CMP–sialic acid to galactose-containing substrates in an alpha 2,6 linkage	Thyroid and brain	(81)
ST6GALNAC1/St6galnac1	ST6 N-acetylgalactosaminide alpha-2,6-sialyltransferase 1		Colon and small intestine	(82)
ST6GALNAC2/St6galnac2	ST6 N-acetylgalactosaminide alpha-2,6-sialyltransferase 2		Skin and testis	(19, 20)
ST6GALNAC3/St6galnac3	ST6 N-acetylgalactosaminide alpha-2,6-sialyltransferase 3		Thyroid, kidney and fat	(83, 84)
ST6GALNAC4/St6galnac4	ST6 N-acetylgalactosaminide alpha-2,6-sialyltransferase 4		Bone marrow and spleen	(84, 85)
ST6GALNAC5/St6galnac5	ST6 N-acetylgalactosaminide alpha-2,6-sialyltransferase 5		Lungs and brain	(86)
ST6GALNAC6/St6galnac6	ST6 N-acetylgalactosaminide alpha-2,6-sialyltransferase 6		Colon and fat	(87)
ST8SIA1/St8sia1	ST8 alpha-N-acetyl-neuraminide alpha-2,8-sialyltransferase 1		Brain, adrenal	(25, 88)
ST8SIA2/St8sia2	ST8 alpha-N-acetyl-neuraminide alpha-2,8-sialyltransferase 2	Catalyzes the transfer of sialic acid from CMP–sialic acid to GM3 to produce gangliosides GD3 and GT3 with an alpha 2,8 linkage	Brain and heart	(23)
ST8SIA3/St8sia3	ST8 alpha-N-acetyl-neuraminide alpha-2,8-sialyltransferase 3	Catalyzes the transfer of sialic acid from CMP–sialic acid to GM3 to produce gangliosides GD3 and GT3 with an alpha 2,8 linkage	Brain	(25)
ST8SIA4/St8sia4	ST8 alpha-N-acetyl-neuraminide alpha-2,8-sialyltransferase 4			(89)
ST8SIA5/St8sia5	ST8 alpha-N-acetyl-neuraminide alpha-2,8-sialyltransferase 5		Brain adrenal	(26)
ST8SIA6/St8sia6	ST8 alpha-N-acetyl-neuraminide alpha-2,8-sialyltransferase 6			(24)

Sialic acids are critical in various physiological and pathological processes, including cell–cell interaction (11, 12), protein stability, regulation of immune responses (16), pathogen recognition and infection, cell migration, and cancer progression (49, 65, 97). There are several reported cases where abnormal expression of sialyltransferases has led to aberrant expression of sialoglycans on glycoproteins, influencing various pathological conditions (98, 99), such as sialuria and hereditary inclusion body myopathy (HIBM). The latter is caused by mutations in the bifunctional GNE enzyme (UDP-N-acetylglucosamine 2-epimerase/N-acetylmannosamine kinase), which plays a crucial role in the biosynthesis of sialic acids (100). Following the sialylation of glycolipids and glycoproteins, they are released into the lysosome by sialidases and then to the cytosol for recycling or broken down by Neu5Ac lyase into ManNAc and pyruvate (101). Mutation in neuraminidase 1, NEU1 (sialidase 1), a lysosomal enzyme that plays a crucial role in the catabolism of sialo-glycoconjugates, leads to lysosomal storage disorder sialidosis, while abnormal NEU1 activity has been implicated in cancer progression, inflammation, and immune response (102, 103). Aside from lysosomal sialidase NEU1 (102), other human sialidases have been identified to include the cytosolic sialidase NEU2 (104), the plasma membrane-associated sialidase NEU3 (105), and mitochondrial membrane-associated sialidase NEU4 (106) (see **Figure 1**). Overall, this highlights the critical role of proper sialic acid biosynthesis and how dysregulation of this process at any step can lead to a wide variety of diseases and a more malignant phenotype in cancer (43, 107).

## Extrinsic sialylation

In addition to the classic intracellular pathway of sialylation, there is new evidence to suggest that sialyltransferases can “extrinsically” catalyze the addition of sialic acid residues outside of the cell membrane (108), and this plays a role in a wide variety of cell processes including cancer progression (109). It has been long known that sialyltransferases (and other glycosyltransferases) exist outside the cell membrane (110), but extrinsic glycosylation was thought to be impossible due to a lack of activated sugar donors. It was not until the Lau Lab demonstrated that the activated sugar donors (29, 30), along with the glycosyltransferases (111) themselves, needed to complete the extrinsic glycosylation reaction could be found in abundance in platelets (29, 112). Subsequent work has implicated extrinsic sialylation in the maintenance of hematopoietic stem cells (113), the production of granulocytes through sialylation of the M-CSF receptor (93, 114), the proper development of B cells in the spleen (115–117), protection against radiation-induced gastrointestinal damage (118), and IgG sialylation (119, 120).

## Neu5Gc and its incorporation into the glycocalyx

It is crucial to note that humans lack the ability to produce N-glycolylneuraminic acid (Neu5Gc) due to an inactivating mutation in the CMAH gene, which is irreversible. CMAH is the only enzyme responsible for the biosynthesis of Neu5Gc in deuterostome (121). Furthermore, note that no human genes have homology to *CMAH*. However, small amounts of Neu5Gc have been found in human tissues, including cancer cells (122–124). This is because humans can incorporate Neu5Gc from dietary sources, especially red meat and dairy products, into their cell surface glycoconjugates (125–127). This incorporation is most prominent in rapidly dividing tissues, such as epithelial cells and carcinomas (127). Once ingested, Neu5Gc is metabolically incorporated into the glycocalyx of cancer cells. The presence of Neu5Gc in cancer cells contributes to changes in their immunogenicity, making them susceptible to interactions with circulating anti-Neu5Gc antibodies (126). Despite the absence of endogenous Neu5Gc synthesis, most humans possess natural antibodies (IgA, IgM, and IgG) targeting Neu5Gc. These antibodies are formed in response to dietary Neu5Gc, making Neu5Gc a Xeno-autoantigen (126, 127). The presence of these antibodies is thought to be a response to dietary exposure or to bacteria scavenging Neu5Gc from the diet and incorporating it into their glycolipids. Anti-Neu5Gc antibodies can contribute to chronic inflammation when they recognize and bind to Neu5Gc present in the human glycocalyx, particularly in cancer cells in a process called xenosialitis, and may promote tumor progression or influence the inflammatory tumor microenvironment (128). Since humans cannot synthesize Neu5Gc, the presence of this non-human sialic acid on cancer cell surfaces renders these cells immunogenic, and become recognizable by the immune system as foreign, potentially leading to immune-mediated destruction of cancer cells. However, chronic inflammation is a well-known driver of cancer progression. The release of inflammatory cytokines such as IL-6, TNF-α, and IL-1β creates a tumor-promoting environment that favors cancer cell survival and growth (129, 130).

## Aberrant sialylation as a hallmark of cancer

For many years, the onset of many cancer types has been recognized to stem from genetic mutations, but more recent emphasis has been placed upon post-translational hallmarks from alterations in biochemical pathways such as sialylation (20, 27, 35, 48, 131–133). Sialylation is a post-translational addition of sialic acid residue to glycoproteins as terminal monosaccharide, which modifies its structure, activity, and longevity and dictates many aspects of a cell’s interactions with the extracellular matrix (ECM) (46, 134). Sialic acids attach in either an α-2,3, α2,6, or α 2,8 linkage to galactose, usually terminating the glycans of glycoconjugates that cover the surface of cancer cells, creating a dense covering of sialylated glycans such as sialyl Lewis-A, -X, sialyl Tn antigen, or the GM2 ganglioside (SLe^A^, SLe^X^, STn, and GM2) (45).

Altered sialylation of several glycoproteins has been implicated in several health issues and diseases including cancer (45, 97, 135, 136). Hypersialylation plays a significant role in cancer development and progression by promoting cancer aggression and metastasis, immune evasion enhancing cancer cell survival, and resistance to therapy (41, 131, 137–139). The increase in α2,6 sialylation of N-glycans is driven by the sialyltransferases ST6GAL1, which is overexpressed in numerous cancer types and are fundamental for tumor growth, metastasis, immune evasion, and drug resistance (27). Overexpression of ST6GALNAC1 in the MDA-MB-231 breast cancer line has also been shown to promote the invasion and migration of breast cancer cells via the EMT pathway (140) while higher levels of *ST8SIA4* promote tumorigenicity in the same cancer cell line (141). Blockade of ST6GAL1 has been shown to inhibit the metastatic spread of prostate cancer to bone (142). Overexpression of α(2-6)-sialic acids in pancreatic adenocarcinoma cell lines mediates increased adhesion to ECM while overexpressed α(2-3)-sialic acids contribute to increased migration (31). In human thyroid cancer, there is an increased expression of sialylated fibronectin (143) and SLe^A^ antigen (144). Adrenal cancer also shows an overall increase in total cellular, cytoplasmic, and total plasma sialic acid content (145) (146). Aberrant upregulation of polysialylation on the neural cell adhesion molecule and serum sialic acids (147, 148) is critical in the progression of pituitary and brain cancer (149) (see **Table 2**). In the brain, there is still limited information on what sialic acid is relatively overexpressed and on which glycan is the sialylation phenotype prominent.

**Table 2:** Sialyltransferase expression in various cancer.

Enzyme	Cancer type (altered/overexpressed)	Effect		References
ST3GAL1	Breast, melanoma, ovarian, and colorectal cancers	Promotion	Cell proliferation, invasion and metastasis, immune evasion, angiogenesis, stemness	(150–155)
ST3GAL3	Colorectal cancers	Promotion	Cell proliferation, invasion, and metastasis	(150, 152)
ST3GAL3	Glioma	Inhibition	Proliferation, invasion and metastasis, immune evasion	(154)
ST3GAL4	Colorectal and gastric cancer	Promotion	Invasion and metastasis, immune evasion	(150, 156)
ST3GAL5	Pediatric leukemia, breast cancer	Promotion	Cell proliferation	(157)
ST3GAL6	Lung and multiple myeloma	Promotion	Invasion and metastasis	(3, 156)
ST6GAL1	Pediatric leukemia, colon, ovarian pancreatic, prostate, colorectal and brain	Promotion	Cell proliferation, invasion and metastasis, stemness	(18, 18, 27, 28, 157–162)
ST6GAL1	Glioma and colon	Inhibition	invasion and metastasis, immune evasion, angiogenesis, stemness	(154, 163)
ST6GAL2	Breast and brain	Promotion	Cell proliferation, invasion and metastasis	(19, 164)
ST6GALNAC1	Breast, prostate, and liver cancers	Promotion	Cell proliferation, invasion and metastasis, stemness	(91, 140, 165)
ST6GALNAC2	Breast cancer	Promotion	Invasion and metastasis	(166)
ST6GALNAC4	Liver cancer	Promotion	Cell proliferation	(167, 168)
ST6GALNAC5	Glioma	Inhibition	Cell proliferation	(169, 170)
ST8SIA1	Breast and melanoma	Promotion	invasion and metastasis, angiogenesis, stemness	(171, 172)
ST8SIA4	Breast and thyroid carcinoma	Promotion	Cell proliferation, invasion and metastasis	(141, 141, 173, 174)
ST8SIA6	Breast, liver, and lung adenocarcinoma	Promotion	Cell proliferation, invasion and metastasis	(175–177)

## Sialic acid mediates immune evasion in tumors; masking selectin and Siglec binding

The presence of sialic acids on cell surfaces influences various cellular behaviors that are crucial for cancer metastasis, including cell adhesion, signaling, and, most importantly, immune evasion. Heavy aberrant O-glycosylation on the surface of mucin residues correlates with metastatic invasion and immune evasion of tumor cells (99, 178–180). Hypersialylated cancer cell surfaces are prime ligands for sialic acid binding lectins (known as Siglecs) on immune cells. The hypersialylated cell surface protein CD24 binds with Siglec-10 on macrophages to prevent tumor cells from undergoing phagocytic death by acting as a “don’t eat me” signal (37). Increased sialyl Lewis antigens on tumor surfaces make the immune system recognize them as migrating leukocytes, not cancer, enabling them to evade the system and colonize other tissues and organs (45). In addition, aberrant expression of sialic acids on cancer cells prevents complement activation, extending longer on the cell surface and serving as a physical barrier to prevent NK cells from accessing their receptors on the cell surface. This, in turn, disables major killing mechanisms of cytotoxic T cells, modulates macrophage function, and dampens dendritic cell activation and function.

**Figure 2:**
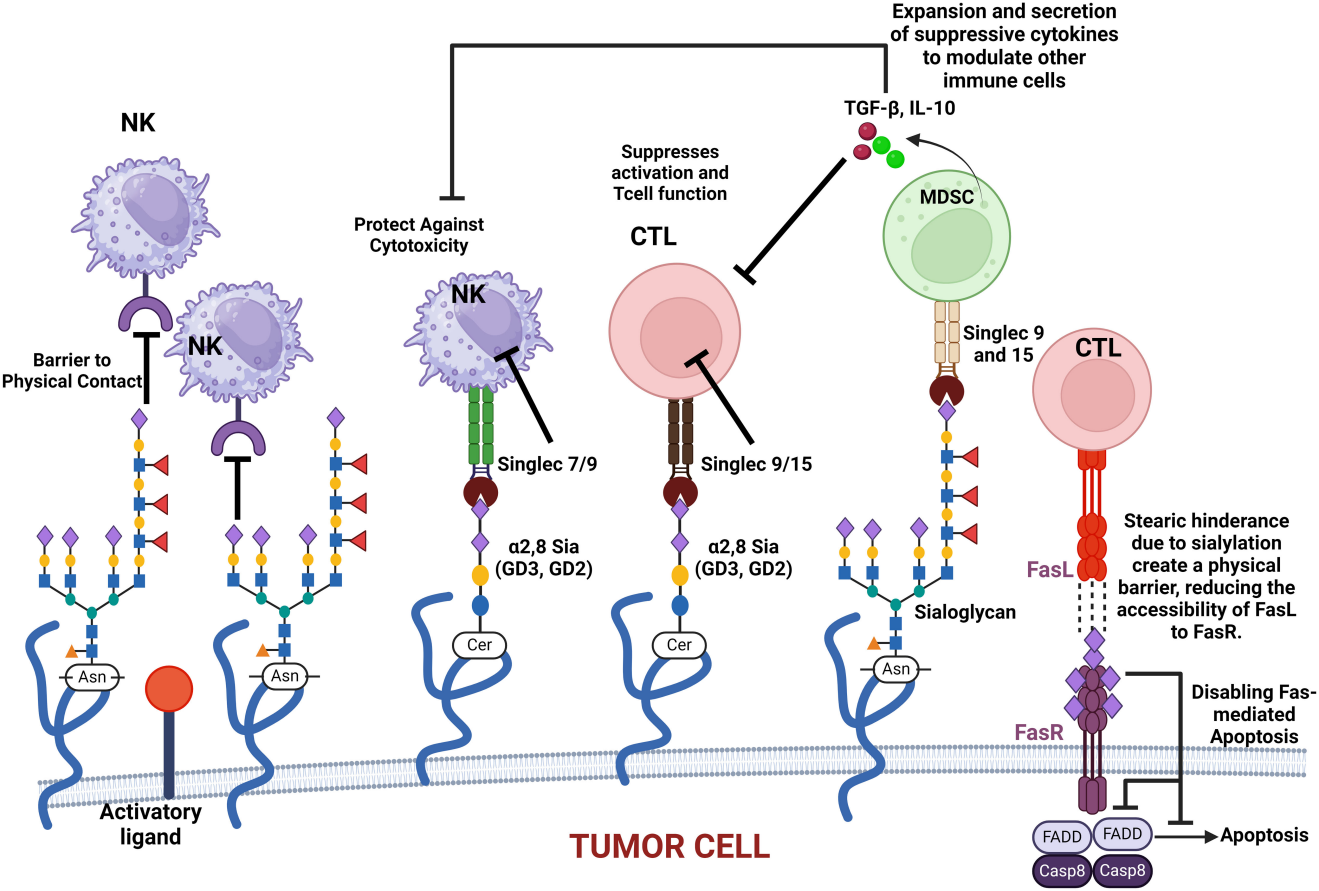
This figure illustrates how various sialylated glycoproteins on the cell membrane interact with immune cell surface components, such as Siglecs, to modulate immune responses. These interactions can protect cancer cells against cytotoxic activity, suppress T-cell activation, and promote the expansion and secretion of modulatory cytokines. Additionally, sialylated glycoproteins create a physical barrier that impedes immune cell contact. The figure also highlights the role of sialylated Fas receptor (FasR) in providing steric hindrance, preventing Fas ligand (FasL) access, and ultimately disabling apoptosis in cancer cells. These mechanisms collectively contribute to immune evasion and cancer progression.

Siglec regulates immune surveillance of cancer, and one of the main results of aberrant sialylation is the loss of Siglec expression in cancer cells, preventing cancer cells from attack by the immune system (33). Siglecs play a critical regulatory role in innate and adaptive immune response via the recognition of mammalian species-specific sialylated glycans (181), as well as regulating cancer immune surveillance (182) (**Figure 2**). Hypersialylation on cancer cells increases sialic acid-binding receptors, aiding immune evasion, and helps to camouflage cancer cells by binding to Siglec receptors on immune cells, transmitting inhibitory signals, and promoting cancer cell survival and proliferation (15, 44). Some Siglecs can also deliver activation signals that enhance antitumor responses, and this interaction affects immune responses, including inflammation (183). Cancer cells have a prominent glycocalyx (35), and they need to evade the NK cells to proliferate, migrate, and metastasize. Siglec-7 and Siglec-9 are inhibitory receptors that bind sialic acid-containing ligands on tumors to dampen the activation of NK cells. Increased expression of Siglec-7 and Siglec-9 ligands on various cancer cells have been shown to decrease their susceptibility to NK cell-mediated killing (36, 184). Siglec-15 on macrophages suppresses the immune microenvironment in PD-L1-negative non-metastatic lung adenocarcinoma (38). It was also reported that inflammatory responses are attenuated or weakened when the activity of sialic acid binding to Siglecs receptors is increased (185). Sialic acid is attached to the outermost glycolipids and glycoproteins on the surface of tumor cells to bind receptors like Siglecs (186).

**Figure 3:**
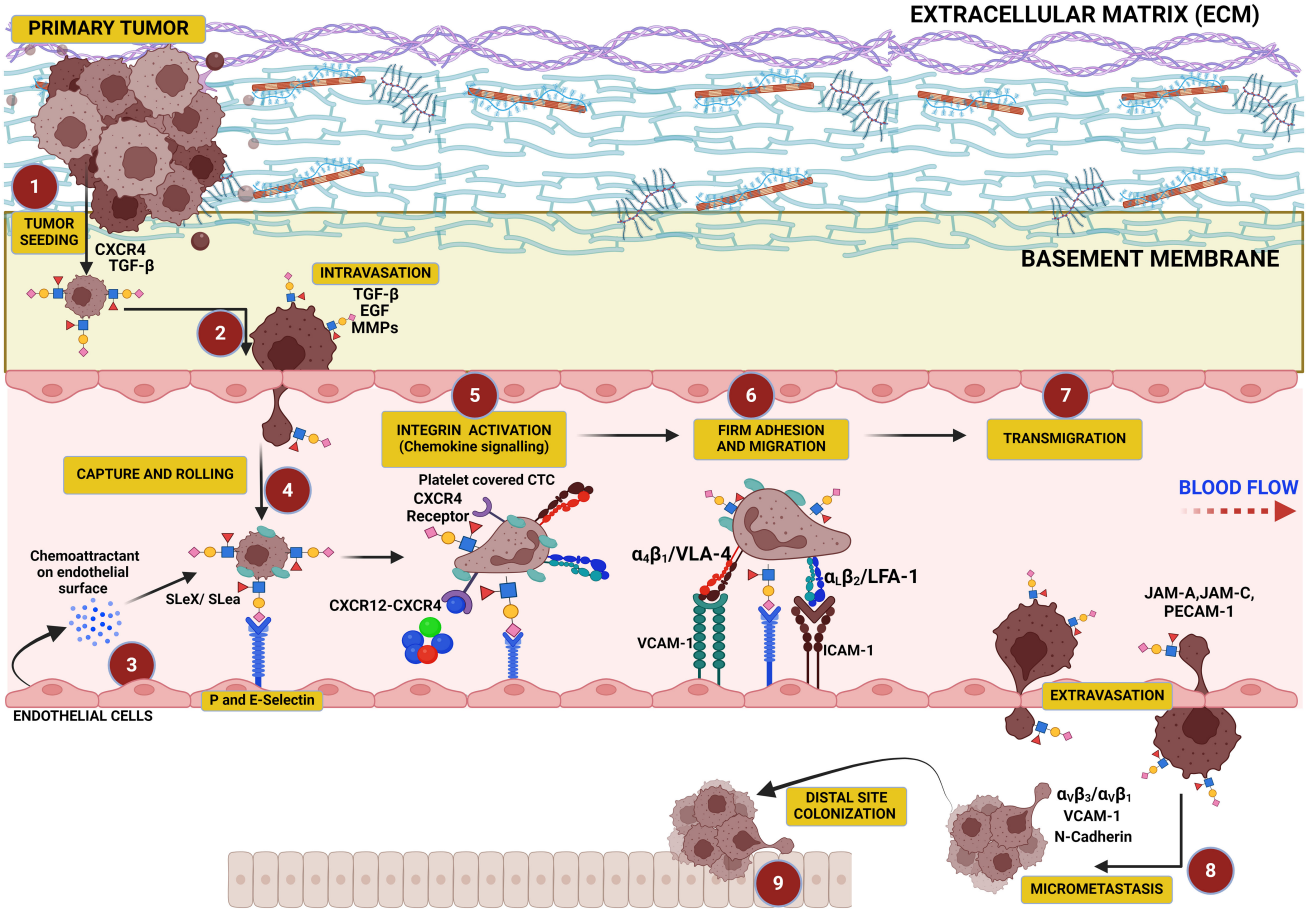
This figure illustrates the critical role of hypersialylation in driving the metastatic cascade. Overexpression of sialoglycans on cancer cells increases local mechanical tension within the primary tumor, leading to cell–cell and cell–matrix repulsion that facilitates tumor cell dissociation. These cells intravasate into the vasculature, where sialyl Lewis X (SLe^x^) on their surface binds to selectins on endothelial cells, mediating their capture, tethering, and rolling along the vascular wall. This interaction precedes cellular migration and transmigration through the endothelium, culminating in colonization at distal sites.

It is critical to note that O-acetylation of sialic acids at position C7, C8, or C9 of sialic acids on the surface of glycoproteins alters their structural conformation and charge, effectively masking the binding of these glycoproteins to key recognition molecules, such as selectins and Siglecs (sialic acid-binding immunoglobulin-type lectins). When sialic acids are O-acetylated, particularly at C9, it sterically hinders or alters the conformation of the sialylated glycans, masking the recognition motifs required for selectin binding. This results in the inhibition of selectin–glycan interaction. When sialic acids are deacetylated, they modulate immune-mediated cytotoxicity via the sialic acid–Siglec pathway (187, 188). Selectins (such as E-, P-, and L-selectins) typically mediate cell adhesion in processes like leukocyte trafficking and cancer metastasis by recognizing sialylated structures like sialyl Lewis X (SLe^X^) (see **Figure 3**). However, O-acetylation at position C9 of sialic acids can block the recognition of SLe^X^ by selectins, reducing cell adhesion and metastasis potential (189). This modification confers a selective advantage to tumor cells by enabling them to evade immune surveillance and promoting their survival and mobility within the body. Similarly, Siglecs, particularly those involved in immune suppression (like Siglec-7 and Siglec-9), depend on the recognition of sialylated glycans. O-acetylation disrupts their ability to bind to these glycans, helping cancer cells escape immune responses that would otherwise be triggered by Siglec–sialic acid interactions.

Overall, O-acetylated sialic acids regulate the dynamics of glycoproteins on the cell surface to influence the formation of glycoprotein lattices and their association into signal-transducing microdomains. By modulating the interaction of glycoproteins with galectins and other lectins, O-acetylation influences how these glycoproteins cluster and move within the plasma membrane to allow for longer retention of sialic acids on the cell surface and, thus, stable glycoprotein networks that aids cellular proliferation, migration, and immune evasion. These microdomains are sometimes referred to as glycosynapses and are very crucial for organizing receptors and other signaling molecules into functional complexes that can transduce signals across the cell membrane.

## Sialic acids’ role as galectin binding modifiers

Galectins are a family of β-galactoside-binding proteins. They modulate various cellular processes, including cell–cell adhesion, immune responses, and tumor progression. It is critical to note that sialic acids play a crucial role in regulating galectin binding to glycoproteins by modifying the exposure of galactose residues. Galectins can be found both inside and outside the cell, with extracellular galectins exerting their functions via an interaction with cell surface oligosaccharides.

Overexpressed sialic acids can inhibit galectin binding by masking galactose residues on the glycan structures. This occurs because galectins preferentially bind to galactose and N-acetyllactosamine (LacNAc) sequences on glycans. When sialic acids cap these sequences, it prevents galectin binding. This sialic acid-mediated inhibition of galectin binding plays a role in cancer progression (190). For example, tumors with high levels of sialylation may avoid galectin-mediated cell–cell interactions, promoting tumor immune evasion and metastasis (191, 192). In contrast, reduction of sialylation can enhance galectin binding, potentially promoting galectin-dependent signaling and tumor cell apoptosis (193).

While there is considerable research interest in galectins, relatively few studies have focused on a critical enzyme that inhibits galectin signaling, namely, β-galactoside α2,6-sialyltransferase (ST6Gal-I). ST6Gal-I catalyzes α2,6-linked sialic acid to the terminal galactose of N-linked glycans, a modification that prevents galectin from binding to β-galactosides, a mechanism for tumor cell survival and immune evasion (190), and this enzyme is highly expressed in various cancer types including colon (194, 195), breast (196), cervical (197), leukemia (157), and brain tumors (198). High levels of ST6Gal-I are strongly associated with increased tumor metastasis and poor clinical prognosis (199, 200). Experimental evidence have shown that α2,6-sialylation of surface receptors by ST6Gal-I prevents Galectin-3 (Gal-3) from binding and initiating apoptotic pathways in epithelial tumor cells (201, 202). Notably, Gal-3, like ST6Gal-I, is also upregulated in various cancers (203–207). This presents a paradox, as upregulation of ST6Gal-I creates a sugar structure that inhibits Gal-3 binding, raising the question of why tumor cells would upregulate both Gal-3 and the sugar structures that block its interaction. To explore this paradox, recent studies have forced the overexpression of ST6Gal-I in SW48 cells (a colon epithelial cell line deficient in both α2,3- and α2,6-sialyltransferases) and examined the effects of recombinant Gal-3 exposure on apoptosis (208) and their results demonstrate that ST6Gal-I-mediated α2,6-sialylation provides a survival advantage to tumor cells by inhibiting Gal-3-induced apoptosis, underscoring the enzyme’s role in tumor progression and resistance to immune-mediated cell death.

## Sialic acid as a cancer biomarker

Sialylation levels and patterns are altered during cancer progression, indicating the potential of sialylated molecules as cancer biomarkers (33). One contributing factor to increased cancer cell signaling is the presence of sialic acid on the glycocalyx. Sialic acid on glycoproteins and glycolipids is known to mediate cell signaling (1). Multiple emerging studies have shed light on the relationship between the presence of sialic acid on these glycans and a more aggressive phenotype of specific cancers (18, 97, 99, 136, 140, 142, 209–211). Most evidently, sialylated glycans regulate cell transduction pathways through its nature to adhere to neighboring cells (through sialyl Lewis antigens and singles) for direct cell–cell signaling, which serves as an essential function for cancer progression (212). The sialic acid sugar also promotes overall tumor progression, not just at the cellular level, but at other levels that can prevent apoptosis, enhance metastasis, and develop resistance to therapy (209). Because of this, sialic acid and sialic acid binding proteins serve as potent biomarkers for all types of cancer for metastasis or invasive cancer spread. In cancers like glioblastoma, inflammation helps tumor cells invade secondary tissues by breaking down the blood–brain barrier. Understanding these mechanisms highlights the role of glycosylation in cancer and suggests targeting hypersialylated glycans as a potential strategy for more effective anti-cancer treatments (213).

Hypersialylation in cancer can be attributed to several mechanisms such as aberrant overexpression of sialyltransferases, varying sialidase/neuraminidase levels, and substrate availability, which all contribute to the rates of sialyation in cancer cells (214). Overexpression of these enzymes aids cancer cells in escaping apoptosis because of the sialylation of specific receptors that mediate apoptosis, such as the Fas receptor. Sialylation of the Fas receptor inhibits the internalization of Fas, blocking the formation of the complexes required for apoptosis (**Figure 2**) (65, 158). Neuraminidases can cleave sialic acid residues from glycans on the glycocalyx, and the overexpression of NEU 1 and NEU 2, found in lysosomes and the cytoplasm, respectively, has been found to inactivate apoptotic pathways of cancer cells (215). Higher sialic acid substrate availability on the glycans themselves leads to the increased activity of sialic acid production pathways. This alteration in metabolic sialic acid production can lead to increased amounts of sialic acid in cancer cells, which can encourage metastasis (216).

## Sialylation of the cancer glycocalyx mediates aggression

**Figure 4:**
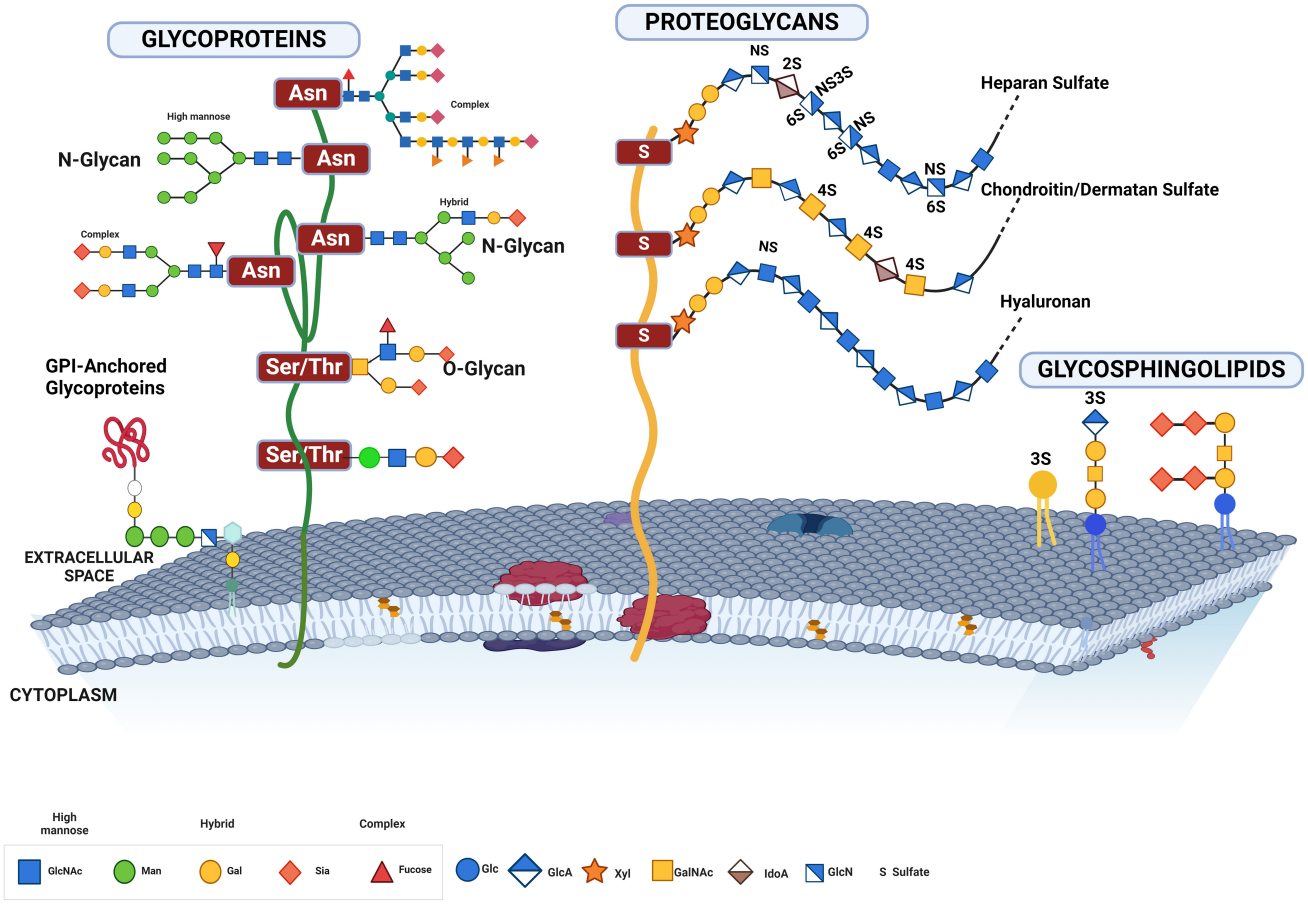
This figure illustrates the various forms of glycosylated proteins present on the cell membrane, highlighting their structural diversity and attachment modes. Depicted are glycoproteins with N-glycans and O-glycans, distinguished by their attachment to specific amino acid residues. N-glycans are attached to asparagine residues, while O-glycans are linked to serine or threonine residues. The figure also includes proteoglycans, which have long, unbranched glycosaminoglycan chains, GPI-anchored glycoproteins, which are tethered to the membrane via glycosylphosphatidylinositol anchors, and glycolipids, which consist of carbohydrate moieties attached directly to ceramide lipids within the membrane. This comprehensive depiction underscores the complex and varied nature of glycosylation on the cell surface, essential for numerous cellular functions and interactions.

In most cancer cells, glycocalyx signatures are usually characterized by upregulated glycosylation (96, 132, 133, 217–221), leading to increased proliferation, migration, and immune evasion as well as invasive capacities (2, 6, 99, 220–222). The size and structure of this glycocalyx are critical not only in defining the tumor cell’s ability to proliferate, migrate, and metastasize but also to evade immune surveillance (8, 99, 223, 224). Three enzymes are crucial in the initiation and/or extension of the three main classes of glycan on the glycoprotein or glycolipids in the cellular glycocalyx. In mammalian cells, *Core 1 β 1,3 Galactosyltransferase-specific molecular chaperone* (*COSMC*) and *Alpha-1,3-Mannosyl-Glycoprotein 2-Beta-N-Acetylglucosaminyltransferase gene* (*MGAT1*) are critical in catalyzing the chain extension of N- and O-linked sugars, respectively, while *UDP-glucose ceramide glucosyltransferase* (*UGCG*) catalyzes the initiation of glycosphingolipid (GSL) sugars, all playing a crucial role in cell signaling and metastasis (138, 225–227). Although the biosynthesis of core glycans is different, chain extensions are similar and often capped by terminal additions of sialic acid and/or fucose (228). Overexpression of sialylated O-glycans is a feature of cancer cell aggression (99), and its knockdown or knockout inhibits tumor growth, invasion, metastasis, and immune evasion (4, 99, 229, 230). Expression of ST8SIA4 in the MDA-MB-231 breast cancer line is associated with breast cancer metastasis (141). N-glycans are also heavily glycosylated in cancer to promote cell motility and loss of contact inhibition (231, 232) and protected against immune responses not only in pancreatic tumors but also in tumors of the lung, ovary, and bladder (233). In the brain, GSLs are implicated in tumor progression (234) as well as in immune evasion (10, 235, 236). Sialic acids are attached to either O-, N-linked glycolipid glycans at their galactose (Gal) or N-acetylgalactosamine (GalNAc) units via α-2,3 or α-2,6 bonds, or to other sialic acid moieties via α-2,8 or α-2,9 bonds (**Figure 4**) by specific sialyltransferases depending on the bond (47, 49).

## Hypersialylated glycans mediate prolonged survival in cancer

**Figure 5:**
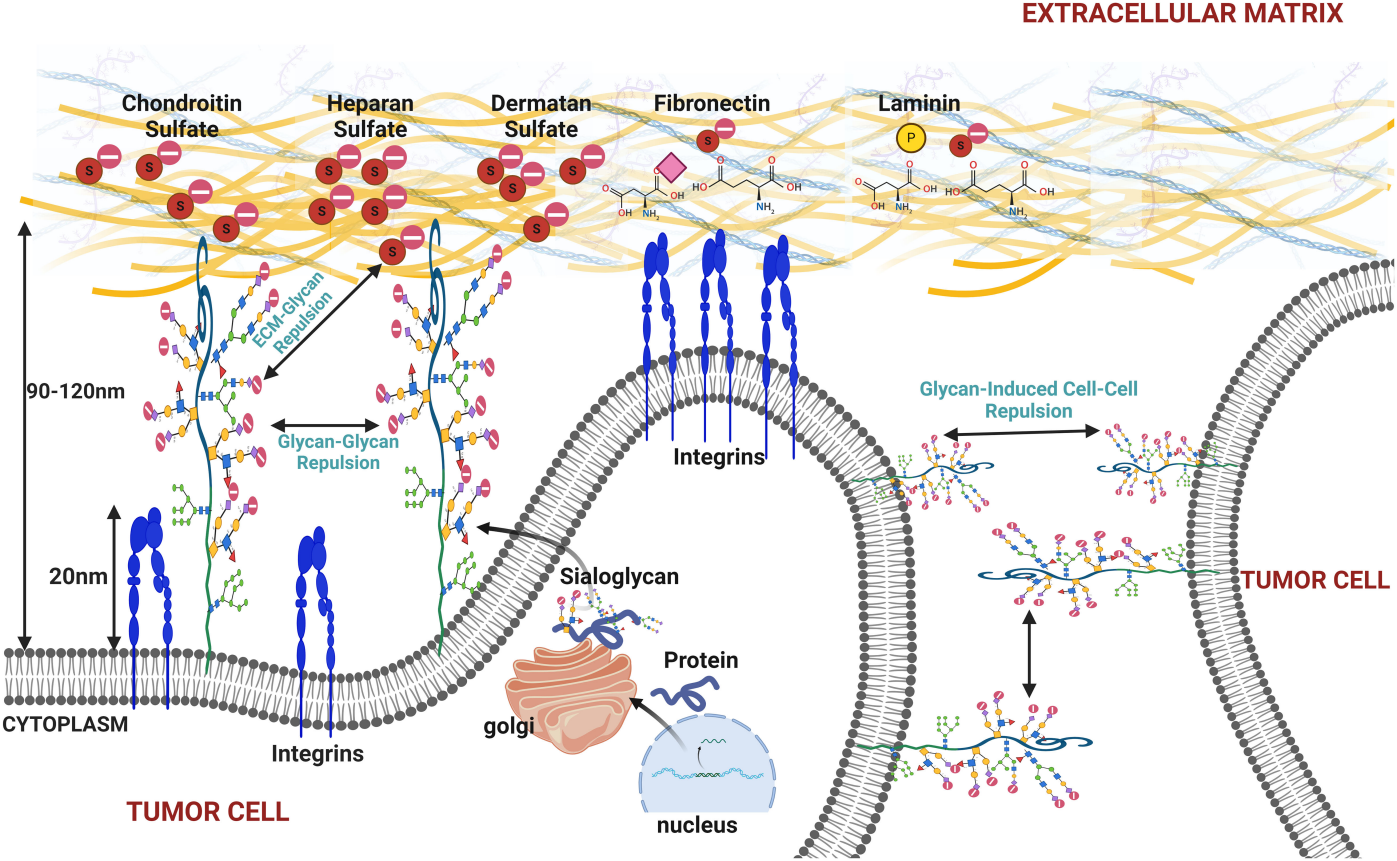
This figure illustrates the effects of increased sialylation on the cell membrane under cancerous conditions, highlighting how heightened sialylation can promote membrane bulging, cell detachment from the extracellular matrix (ECM), and tumor cell disaggregation. The figure depicts the repulsive interactions between sialylated glycoproteins on the cell membrane, causing glycan–glycan repulsion and resulting in membrane bulging. Additionally, it shows how sialylated glycans interact with the ECM components and neighboring tumor cells, leading to glycan–ECM and glycan-induced cell–cell repulsion. These mechanisms contribute to cancer cell detachment and increase metastatic potential, emphasizing the role of sialylation in cancer progression and metastasis.

Cancer cells often have high levels of sialylation (237), which are often associated with malignancy and poor prognosis in patients (94, 95). Increased sialylation can increase local negative charges (as sialic acid is the only monosaccharide to carry a charge) on the cancer cell membrane to physically disrupt cell–cell adhesion and promote detachment from the tumor mass through electrostatic repulsion, which ultimately leads to membrane bending (13) (**Figure 5**). In N-glycans, the mannosyl (alpha-1,3-)-glycoprotein beta-1,2-N-acetylglucosaminyltransferase (MGAT) family of enzymes including MGAT1, MGAT4A, and MGAT5A are upregulated in many cancers, to fuel the loss of contact inhibition, increased cell motility, invasion, and metastasis (42, 233, 238–243). MGAT1, MGAT2, MGAT4, and MGAT5 sequentially add N-acetylglucosamine (GlcNAc) residues to the core mannose residues of N-glycans, resulting in highly branched complex and hybrid N-glycans. Increased activity of MGAT enzymes leads to more complex and branched N-glycan structures on glycoproteins to provide more sites for sialyltransferases to add sialic acid residues. Increased branching structures facilitate the attachment of multiple sialic acids, leading to hypersialylation.

In O-glycans, COSMC is a specific molecular chaperone required for the proper folding and function of the enzyme C1GALT1. In cancers, dysregulation of COSMC has been reported to cause the accumulation of Tn antigen to form the sialyl-Tn antigen. Dysregulated COSMC causes T-synthase (C1GALT1) to be misfolded and degraded, leading to aberrant glycosylation (244, 245). Mucin overexpression in epithelial cells increases the number of glycosylation sites to increase sialylation and fuel resistance against NK cells (99). Overexpression of COSMC in human colon cancer cells significantly enhances cell migration, invasion, and cancer survival (246–248).

In cervical and breast cancer, overexpression of UDP-glucose ceramide glucosyltransferase (UGCG) led to increased synthesis of glucosylceramide and subsequently more complex GSLs to fuel cell proliferation and glycolysis via the PI3K/AKT pathway (7, 9, 249). The increased availability of precursor molecules facilitates the synthesis of gangliosides, which are often hypersialylated in cancer cells. GSLs are expressed in the brain with a bulk of sialic acids to form gangliosides (250–252), and their aberrant expression drives tumor growth and survival (252–254).

## The composition of the cellular glycocalyx modulates membrane dynamics in cancer

The most consistent finding in glycobiology-based cancer research is the priming of cell surface with a robust sugar glycocalyx to mechanically foster cell growth and survival. Upregulation of glycoproteins in cancer is a recurrent event (138, 225–227) to enhance integrin-dependent cell growth and survival (228, 255) and promotes a mesenchymal-like phenotype (217). In many cancer types (breast, brain, lungs, and prostate), glycosylation events are increased to enable addition of more (bulkier) sugars on the glycan including the terminal sialic acid being the most important. More “bulky” glycan structures on the glycocalyx components help to mechanically apply tension the cell membrane and provide local repulsive forces within the membrane vicinity and between adjacent cells, leading to membrane bulging and cell dissociation from matrix and from other tumor cells. These more bulky glycans in many types of cancer extend further (>50 nm) from the cell surface than the integrins (~30 nm) (8), and because of the desirability of most cancer cells to attach to the ECM through their integrins, there is a forced ECM–integrin interaction. This serves to mechanically induce a feedback pull on both the ECM and the nucleus of the tumor cell and subsequently promote the activation genes involved in cancer cell proliferation and metastasis (14, 159, 217, 256–261). Engineering of O-glycans on the cancer glycocalyx demonstrated that a thick and dense glycocalyx could trigger complete cellular detachment from the ECM to facilitate prolonged cell survival (236). Several research lines have applied gene editing to affect the enzymatic function of specific precursor sugars of the entire glycan tree to make cancer cells susceptible to immune attack (262–265).

The cancer cell glycocalyx is heavily decorated by glycan structures (266), and this glycan bulk induces an upregulation of both mesenchymal and bulky glycocalyx-related genes to drive aggression (217). A bulky glycocalyx have also been shown to drive metastasis by increasing cell cycle progression (8). Other reports showed that an increase in the size of the glycocalyx with the MUC1 ecto-domain was sufficient to drive metastatic potential in an *in vivo* model of breast cancer (8) as well as promoting immune evasion in epithelial cells that had increased MUC1 expression (99). Recent reports further revealed that increased MUC1 expression is a precursor to hypersialylation and immune evasion. Overexpression of mucins in the MCF10A epithelial cell line increases the glycocalyx bulk to evade immune detection while its knockout abrogated the glycocalyx bulk in the ZR-75-1 breast cancer cell line (99).

As our knowledge in this area continues to expand, it has become increasingly evident that the glycocalyx of tumor cells is closely associated with its ability to migrate (267–270). A tumor cell’s glycocalyx serves to tension their membrane and enhance integrin clustering and activate downstream pathways involved in tumor proliferation and invasion (8, 223, 271, 272), as well as extravasation (17, 273, 274). Other studies indicated that mucin degradation in ZR-75-1 breast cancer cell line led to an increased NK-mediated cytotoxicity (99) while genetic disruption of the glycocalyx in melanoma cells tends to be anti-metastatic (275). Recently, synthetic mucin glycopolymers of low and high densities were expressed on epithelial cell membrane to increase the relative size and density of mucin and modulate plasma membrane dynamics (236). Glycocalyx-mediated tensioning has been identified in many cancer types including glioma cells, which cause glioblastoma multiforme (GBM) to adopt a more mesenchymal and lethal phenotype with high migration velocity (217). This finding is consistent with other studies where increase in mucin glycosylation (O-glycan) correlates with metastatic invasion and immune evasion (99, 178–180).

## Sialylation-induced mechanical tension modulates signaling in cancer

**Figure 6:**
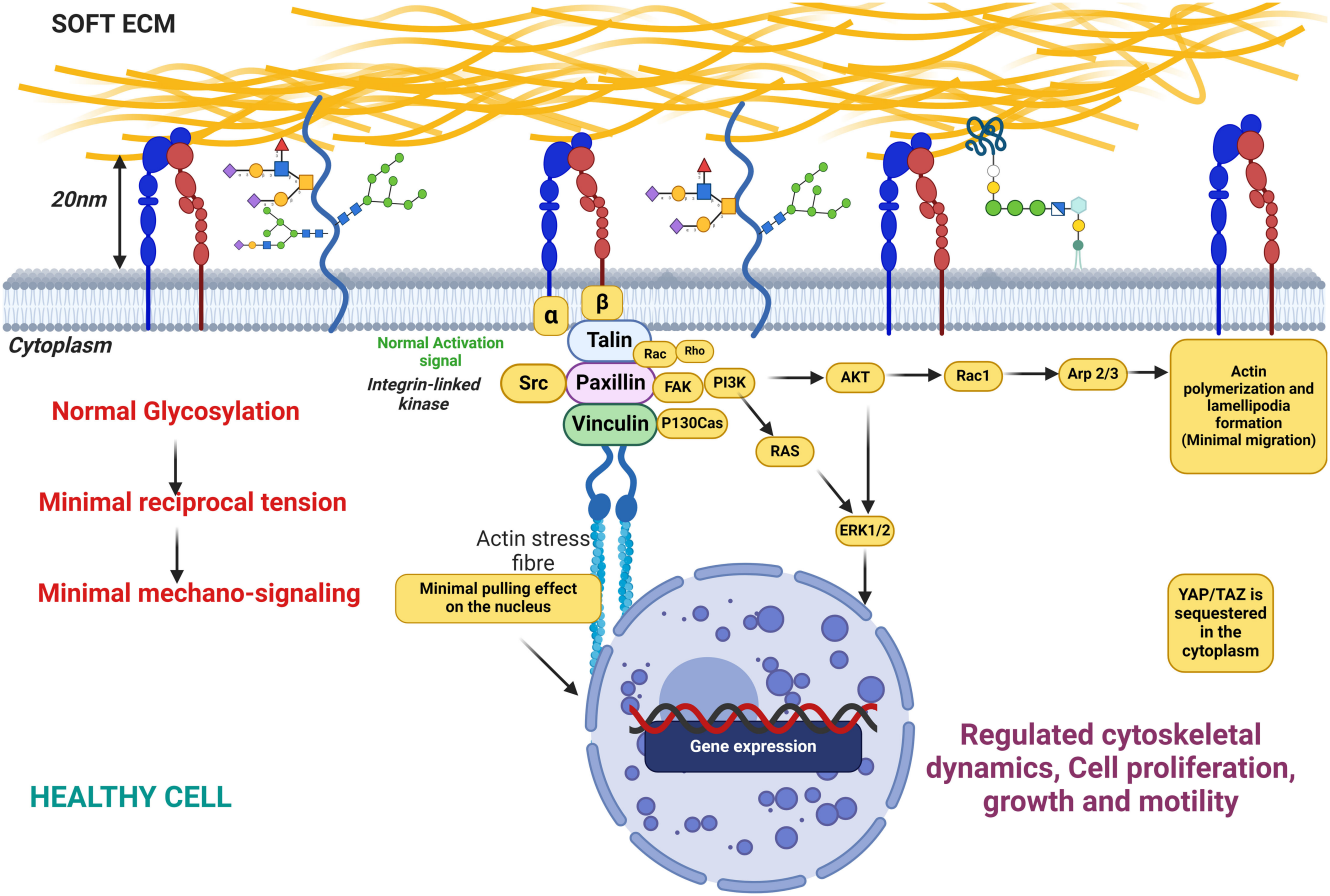
This figure illustrates the state of cellular glycosylation on the membrane under healthy conditions, characterized by minimal reciprocal membrane tension and low mechanosignaling activity. The figure also depicts the distribution and structure of glycosylated proteins on the cell membrane, highlighting the role of glycosylation in maintaining cellular functions in a healthy physiological state. Key components include glycoproteins, the actin cytoskeleton, and signaling molecules, demonstrating their interplay in preserving normal cellular behavior with minimal mechanical stress to the membrane.

Physical stress induced on the cell membrane by glycan capped by sialic acid can impact cellular behavior to reorganize and activate surface receptors’ integrins. This tension-activated integrin pulls on the ECM ligands, causing a tension-mediated glycocalyx–integrin feedback loop to promote mesenchymal-like phenotype in most cancer cells that overexpresses mucin glycoprotein. This, in turn, has a pulling mechanical effect on the nucleus to promote downstream signaling with growth factor receptors, a positive factor to G1 cell cycle progression (217, 223, 276). Overexpression of mucin glycoproteins in cancer cells potentiate more glycosylation hotspots for O- and N-glycosylation, which are often capped with an excess of sialic acid and/or fucose at the terminal end of the sugar glycan. Increased expression of mucin in MCF-10A breast epithelial cell line correlates with sialic acid expression (99) while increased sialic acids on glycoproteins have been reported to stimulate not just integrin-FAK mechanosignaling (8, 217) (**Figure 6**). Sialylation events such as overexpression of ST6GalNAcII mediates the invasive properties of breast carcinoma through the PI3K/Akt/NF‐κB signaling pathway (166). ST6GALNAC1 expression has been shown to promote the invasion and migration of breast cancer cells via the EMT pathway (140).

Other studies have shown that MUC1 is heavily sialylated and mice deficient in MUC1 resist tumor formation (277) while primary tumor xenografts that overexpress MUC1 grow and metastasize more aggressively (268). The implication of this finding is that tumors with bulky glycans on their mucins may foster tumor progression and aggression. Heavily sialylated mucins have been reported to bend the cancer cell membrane and stimulate integrin-FAK mechanosignaling. This occurs due to the negatively charged sialic acids populating the membrane, creating a repulsive effect in the local vicinity of the cancer cell membrane and pulling all the integrins and other surface receptors apart. As a result, focal adhesions are formed and the receptor integrins are forced to bind and pull on the ECM as well as the intracellular cytoskeleton to mediate a stiffer ECM. The subsequent autophosphorylation of tyrosine pY397-FAK assembly, which is an activation signal to other downstream mechanosignaling events involved in cell migration, proliferation, and aggression (8, 217, 223, 261), then occurs. FAK assembly disseminates adhesion and tensional information from focal adhesions to the rest of the cell via autophosphorylation at tyrosine 397, as well as increased phosphorylation at tyrosine 925 (278). The cell senses increased substrate stiffness through integrins to induce assembly of focal adhesions and further cause activation of focal adhesion kinase (FAK) (34, 279). This leads to enhanced MAPK activation, such as Akt phosphorylation, which then promotes G1 cell cycle progression through expression of proteins like the cyclins (8). The AKT pathway and MAPK pathway can influence each other’s activity through crosstalk and regulatory interactions. AKT can directly or indirectly modulate the activation and function of various MAPKs, including ERK1/2, JNK, and p38 MAPK. Staining for pY397-FAK, cyclin D1, and pAKT substrate demonstrated that overexpression of the MUC1 ectodomain increases mechanosignaling, cell cycle progression, and MAPK activity (280).

Taken together, increased sialylation drives integrin clustering through kinetic funneling, leading to increased signaling from focal adhesion-associated proteins such as FAK in combination with growth factor signaling.

## Altered sialylation in brain cancer

Owing to the brain’s importance in controlling the central nervous system and overseeing most of the body’s functions, tumor growth in the brain has unique cell types, immune context, anatomy, and metabolic limitations as the tumor microenvironment of the brain is significantly different compared to other parts of the body. This unique microenvironment, protected by the blood–brain barrier, favors low rate of metastasis out of the brain while favoring higher rate of metastatic invasion within the brain (281, 282). How brain tumors manage to navigate this microenvironment from other organs presents unique clinical challenges especially in patients with lung, breast, and skin cancer that has metastasized to the brain (282, 283).

In normal tissue, sialic acids (gangliosides and polysialic acids) maintain the structural stability of stem cells and its self-renewal. In the brain, gangliosides influence stem cell proliferation and differentiation pathways while the interaction of polysialic acid with neural cell adhesion molecules (NCAMs) modulates cell–cell interactions that are essential for the maintenance of neural stem cells, thereby promoting both their self-renewal and the regenerative processes (28, 252, 284). In glioblastoma, aberrant expression of sialic acids plays a significant role in the enhanced self-renewal and survival of circulating stem cells (CSCs). CSCs are distinguished by altered glycosylation patterns, particularly in sialylated glycoproteins. These modifications promote the ability of CSCs to evade immune detection (**Figure 2**) and resist therapies, which is a critical aspect of their aggressiveness. Sialic acid-rich gangliosides contribute to maintaining the “stemness” of these cells by stabilizing key signaling molecules on their surface, to facilitate self-renewal and tumorigenesis (284). Additionally in glioblastoma, ST6Gal1-mediated α2,6-sialylation of crucial receptors such as EGFR and TNFR1 has been shown to enhance tumor-initiating cell (TIC) properties to foster increased self-renewal, therapy resistance, and invasion. This heightened level of sialylation supports cancer stem cell (CSC) survival by stabilizing cell surface proteins that drive oncogenic signaling (28). Ganglioside GD3 are highly expressed in CSCs to facilitate self-renewal and invasiveness. GD3 has been identified as a marker for glioblastoma stem cells (GSCs) and overexpression of ST6Gal1 and GD3 synthase (GD3S) has been shown to enhance CSC properties by stabilizing oncogenic signaling pathways like c-MET signaling (252).

Research on glycosylation in brain cancer, particularly GBM, is limited but suggests a potential role for α2,6 sialylation and the enzyme ST6GAL1 in promoting GBM growth. Elevated α2,6 sialylation levels correlate with increased GBM cell growth and self-renewal. ST6GAL1 was identified as a key regulator of α2,6 sialylation in GBM, with higher expression observed in brain tumor-initiating cells (BTICs) (28). Knockdown of ST6GAL1 in BTICs resulted in reduced growth, self-renewal capacity, and tumorigenic potential (18). Additionally, ST6GAL1 was found to modulate the levels of key BTIC regulators, such as PDGF receptor β (PDGFRB), activated leukocyte cell adhesion molecule, and neuropilin, through its regulation of α2,6 sialylation. These findings underscore the importance of ST6GAL1-mediated α2,6 sialylation in driving GBM growth (18, 40).

Other families of sialyltransferases have been identified in glioblastoma and other types of brain cancer including other ST6GAL family (160–162, 285), ST3GAL Family (3, 286, 287), and ST8SIA Family (88, 286, 288, 289). However, it is still unknown what proteins and lipids of the cellular glycocalyx present these critical sialylated glycans, which are the most important functional end groups that potentiate aggressive phenotype in glioblastoma.

## Glycocalyx bulk mediates a stiffer ECM to promote cancer cell migration

**Figure 7:**
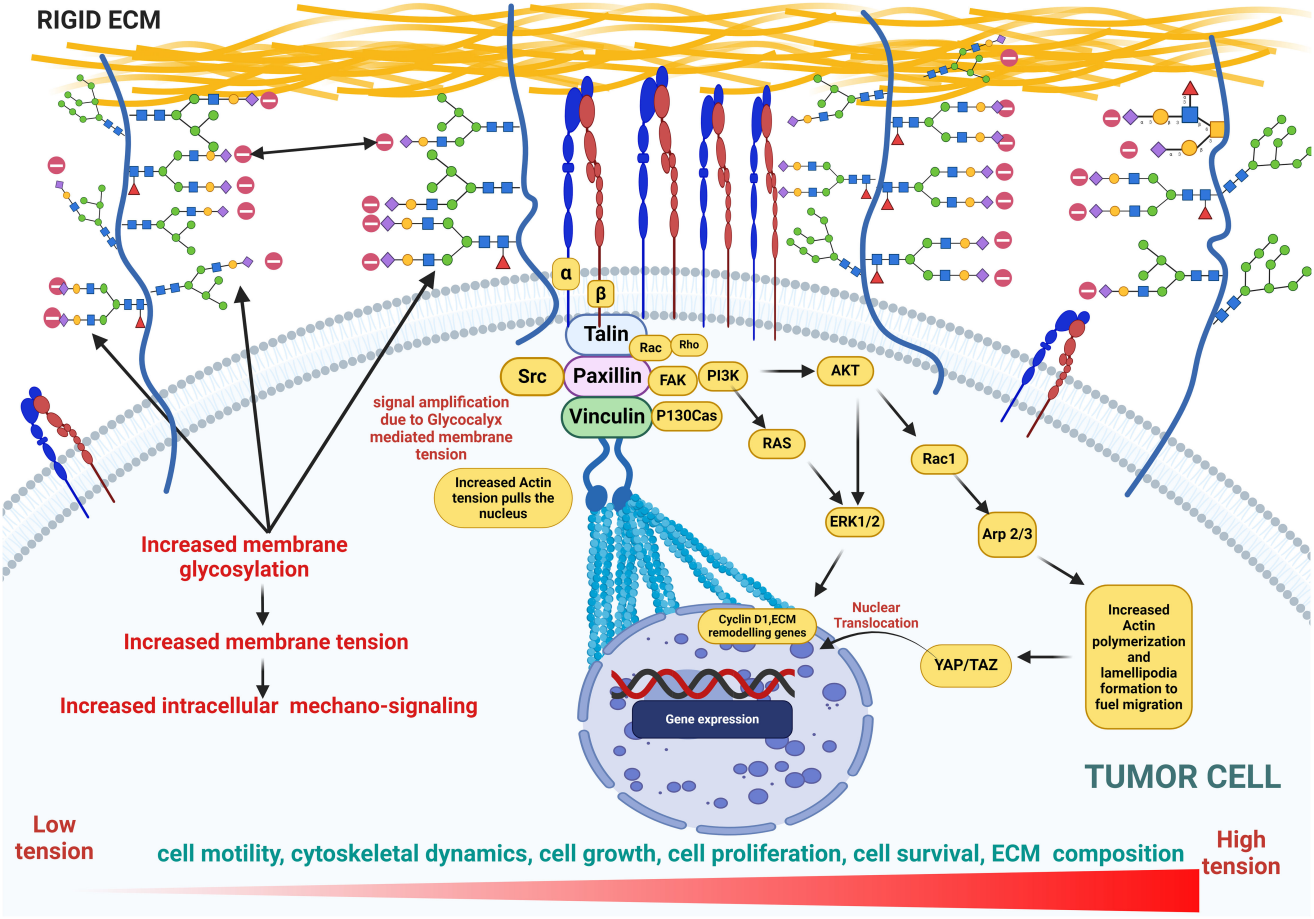
This figure illustrates cellular glycosylation on the membrane under cancerous conditions, highlighting the role of glycocalyx in promoting membrane bulging as well as increased membrane tension and mechanosignaling. This tension drives an upregulation in the expression of genes involved in cytoskeletal dynamics, actin polymerization, cell proliferation, growth, and motility, emphasizing the pathological shifts in cellular behavior. Key features include the distribution of glycoproteins, amplified signaling pathways, and the resulting structural and functional changes in the cytoskeleton, underscoring the impact of glycosylation on cancer progression.

Aside from sialylation serving to bulk the cancer cell’s glycocalyx, it also causes the glycocalyx to extend further from the cell membrane. In certain cancers, the glycocalyx can extend longer than the integrins away from the cell membrane. This causes a forced protrusion of integrin binding to the ECM, leading to a “push and pull” on both the ECM and the nucleus, respectively (223, 271, 290). This pulling effect induces a physical change in the tumor microenvironment to cause physical stiffening of ECM, increased cellular contractility, and substrate tensioning (261). As such, cells continuously sense and modify their behavior in response to this physical cue via mechanotransduction to activate integrins that bind the ECM to ultimately modify the migratory behavior of cells (291, 292) (**Figure 7**). The pulling effect of integrins on the ECM mechanically activates tenascin-C (TNC) that is produced and secreted onto the ECM to further increase ECM stiffness, cellular griping, and contractility to increase traction. This event increases the migratory behavior of the cell, and this is true for most cancers including breast and brain cancer (217, 293, 294).

The stiffness of the ECM dictates how sialic acids influence cellular physiology. Softer ECM support stem cell self-renewal and tissue regeneration. This ECM softness allows for less rigid cell adhesion to reduce integrin signaling mediated by FAK. Sialic acids, particularly in the form of glycosylated proteins like gangliosides, help maintain stem cell function by modulating integrin activation and reducing over-activation of mechanotransduction pathways (239, 295). In cancer, a stiffer matrix predominates to promote CSC invasion and migration (296). Studies show that stiffer ECM, with higher collagen content and rigidity, activates SRC/FAK signaling, which is crucial for CSC migration, invasion, and metastasis. Sialic acids, particularly those catalyzed MGAT5, are crucial in the N-glycosylation of integrins, to enhance their interaction with galectins and promote focal adhesion turnover and mechanosensing. This is essential for cancer cell migration and invasion in stiffer ECM (239, 295). For example, GSCs exhibit maximum migration at an optimal matrix stiffness (166 kPa) due to MGAT5-dependent N-glycosylation (239).

In GBM, stiffness of ECM is altered due to elevated TNC to promote growth, survival, migration, and invasion (295, 297). An increase in the ECM stiffness compromises the cell’s vascular integrity to induce the expression of epithelial-to-mesenchymal (EMT) transition genes (298, 299). A growing body of research also reveals the positive role of sialic acid bulk on the glycoprotein in mediating stiffer ECM to promote cell proliferation (223). Stable expression of sialyl-Tn antigen in the T47-D human breast cancer cell line induces a decrease of cell adhesion and an increase in cell migration (211). Direct alteration of brain stiffness resulted in aberrant axonal growth and migration (300). Barnes et al. (217) also reported that proneural GBMs had longer migration trajectories and higher migration velocities on stiff PA gels. TNC expression has been reported to increase proliferation of neurogenic precursors in the developing ventricle (301). Miroshnikova et al. (295) reported that reduced TNC expression led to a fourfold decrease in the stiffness of ECM and lower tumor migration in GBM. Cells exposed to chronically stiffened ECM undergo EMT, promoting their switch to a motile state with increased aggression and migration (217, 302). Enhanced ECM stiffness also drives GBM cell proliferation and a phenotype reminiscent of EMT, which further enhances GBM invasion (32, 303). In all, a hypersialylated glycocalyx is a potent mechanosensor known to drive an increased stiffness to potentiate migratory phenotype in brain cancer.

## A sialylated glycocalyx is indispensable to the cancer metastatic cascade

The overall charge of the ECM is typically negative as this arises from several components of the ECM, including proteoglycans (covered in glycosaminoglycans or GAGs) such as heparan sulfate, chondroitin sulfate, and dermatan sulfate, which are highly negatively charged due to the presence of sulfate groups and carboxyl groups (304). The glycoproteins on the ECM such as fibronectin and laminin also contribute to the negative charge through their carbohydrate side chains, which may include sialic acid residues that carry a negative charge (305, 306). Moreover, increased sialylation on integrins can induce detachment from the ECM, promoting cancer cell migration and tissue invasion (307–309) (**Figure 3**). The stearic repulsion contributed by negative charges on the ECM and negatively charged sialic acid on the cancer cell surface fuels the detachment of cancer cells from the tumor mass and subsequent metastasis. The detached tumor cells then exit the vasculature where they are recruited to the endothelium via selectin ligands for an onward transmigration through the endothelium to form secondary metastases (310, 311). Cancer cell surfaces are enriched with glycans capped with SLe^X^ and SLe^a^ oligosaccharides that can interact with selectins in the endothelium, promoting cancer cells to adhere to, migrate along, and extravasate through the endothelium (33).

The progression of cancer is strongly related to sialylated glycans and their interaction with selectins, which interact with SLe^X^ moieties on the cancer cell glycocalyx and are critical for cancer cell tethering and rolling on the vascular endothelium upon intravasation and in the direction of shear flow (**Figure 3**). This initial interaction of SLe^X^ and either P- and E-selectin is important for transmigration along the endothelium and subsequent steps in cancer metastatic cascade and their extravasation to distal sites, which is similar to leukocyte adhesion cascade (312, 313). Several studies have shown that in the absence of the full SLe^x^ moiety, the initial recruitment of leukocytes could not be initiated, which may also be true in circulating tumor cells (CTCs) (150, 314, 315) as well. Furthermore, excess sialylation in cancer induces EMT phenotype including cell–cell detachment from primary tumor, ECM invasion, and distal site colonization following metastasis. E-selectin ligands are reported to play a role in the metastasis of cancer cells to bone (316) by inducing EMT and WNT signaling (317). Overall, this demonstrates that when EMT is induced in cancer cells, selectin-binding SLe^X^ oligosaccharides are upregulated and promote the invasion of cancer cells.

## Sialylation as a potential target for early diagnosis and therapeutic intervention

Given the importance of sialic acids in the progression of cancer, some enzymes are potential targets for drug development including sialic acid synthases, CMP–sialic acid synthetases, sialyltransferases, sialidases, and sialic acid modification enzymes (48). Early diagnosis and treatment are critical in managing cancer; thus, the need to understand the formation and pathological alterations of its progression is of upmost importance. Hypersialylation of glycoconjugates has been implicated as an important disease biomarker and a potential therapeutic target (318). In many endocrine disorders, sialylation has been reported as an important disease biomarker as there is increased sialylation in cancer patients compared to healthy patients, which is also directly proportional to the cancer stage (318, 319). New evidence has also demonstrated that de-sialylation repolarizes tumor-associated macrophages (TAMs) (320), and treatment with neuraminidase to cleave sialic acid residues or its inhibition using 3Fax-Neu5Ac enables activation of natural killer (NK) cells to kill multiple myeloma cells (210, 321). Recent reports also point to sialic acid–Siglec interactions as new immune checkpoints to be targeted in order to achieve adaptive and innate antitumor immunity (39, 320). This remains an active area of investigation as the complicated and broad sialoglycan–Siglec expression pattern in the immune system (320) and the elucidation of all of the Siglec ligands have not been fully characterized to date (65). ST6Gal-I overexpression in tumors functions as a crucial modulator of tumor cell survival, particularly by disrupting galectin–glycan interactions that would otherwise promote apoptosis. This mechanism, combined with the enzyme’s involvement in cell adhesion and metastasis, makes ST6Gal-I a significant target for therapeutic intervention in cancer treatment.

Glycans on cell surface glycoconjugates are aberrantly expressed in tumor cells, which consequently gives them a unique glycan signature as compared to healthy cells (322). With cell surface glycans currently being targeted by therapeutic agents to aid treatment and for diagnostic purposes (65), the identification of the specific glycan composition of cancer cells will be of great importance to understanding the role of glycans in immune evasion (45, 322, 323). It was suggested that tumor cells have specific glycan signatures (“Glyco-codes”) that, if deciphered, can be considered a novel immune checkpoint that offers new therapeutic opportunities (322). Using antibody–NEU conjugates has shown promising results in precision glycocalyx editing for immune therapy (45). This method uses a specific antibody to drive NEU to selectively cleave sialic acids from tumor cells exposing de-sialylated (and galactose exposed) cancer cells to immune cells’ attack (324). This same approach showed great promise in breast cancer treatment (99, 325). Another approach that targets sialic acid glycans in cancer immunotherapy with great potential is the use of anti-glycan vaccines, inhibiting cancer-associated glycan lectin interactions and dendritic cell targeting (322).

## Conclusions and future directions

One trend that remains of great importance as it relates to cancer sialylation is determining which specific glycan chains, proteins, and lipids these sialylated glycans reside on. Future work should target the disruption of chain-initiating glycosyltransferases as a promising approach in determining both the most important glycan structure of the glycocalyx that displays the chain terminating sialic acids and which backbone component it rests on. This will determine the critical sialylated glycans mediating aggression in various cancer types so that we can adequately target the glycan for disruption. This could be achieved by truncating either of the chain initiating/extension enzymes for O-linked, N-linked or GSL glycan synthesis (326). In both the healthy and cancer glycocalyx, MGAT1, COSMC, and UGCG are critical chain initiating enzymes that synthesize N-linked glycans (complex and hybrid), O-linked glycans, and GSLs, respectively, and may play a crucial role in cell signaling, immune evasion, and cancer metastasis (138, 225–227). Determining which branch displays the sialylated glycans is the first step toward effectively “pruning” the cancer glycocalyx to be more susceptible to treatment.

Immunoengineering is also a very promising approach as equipping effector immune cells with surface-displayed or secreted enzymes that disrupt the glycan structures displaying the critical sialylated glycans can also represent an attractive strategy for the enhanced killing of tumor cells through protein engineering. Pharmacological agents or metabolic inhibitors could also be exploited to potentially disrupt sialic acid synthesis (99). Unravelling these mechanisms has the potential to accelerate the discovery of therapeutic interventions that overcome the protective sialic acid barrier in cancer cells.

## References

[1] Psefteli PM Kitscha P Vizcay G Fleck R Chapple SJ Mann GE. Glycocalyx sialic acids regulate Nrf2-mediated signaling by fluid shear stress in human endothelial cells. Redox Biol. (2021) 38:101816. doi: 10.1016/j.redox.2020.101816 33340902 PMC7750408

[2] Boscher C Dennis JW Nabi IR. Glycosylation, galectins and cellular signaling. Curr Opin Cell Biol. (2011) 23:383–92. doi: 10.1016/j.ceb.2011.05.001 21616652

[3] Li J Long Y Sun J Wu J He X Wang S. Comprehensive landscape of the ST3GAL family reveals the significance of ST3GAL6-AS1/ST3GAL6 axis on EGFR signaling in lung adenocarcinoma cell invasion. Front Cell Dev Biol. (2022) 10:931132. doi: 10.3389/fcell.2022.931132 36092699 PMC9462654

[4] Ma Z Vosseller K. Cancer metabolism and elevated O−GlcNAc in oncogenic signaling. J Biol Chem. (2014) 289:34457–65. doi: 10.1074/jbc.R114.577718 PMC4263853 25336642

[5] Schnaar RL Gerardy-Schahn R Hildebrandt H. Sialic acids in the brain: gangliosides and polysialic acid in nervous system development, stability, disease, and regeneration. Physiol Rev. (2014). doi: 10.1152/physrev.00033.2013 PMC4044301 24692354

[6] Li W Wu C Yao Y Dong B Wei Z Lv X. MUC4 modulates human glioblastoma cell proliferation and invasion by upregulating EGFR expression. Neurosci Lett. (2014) 566:82–7. doi: 10.1016/j.neulet.2014.02.033 24582898

[7] Wegner MS Schömel N Gruber L Örtel SB Kjellberg MA Mattjus P. UDP-glucose ceramide glucosyltransferase activates AKT, promoted proliferation, and doxorubicin resistance in breast cancer cells. Cell Mol Life Sci. (2018) 75:3393–410. doi: 10.1007/s00018-018-2799-7 PMC11105721 29549423

[8] Woods EC Kai F Barnes JM Pedram K Pickup MW Hollander MJ. A bulky glycocalyx fosters metastasis formation by promoting G1 cell cycle progression. Elife. (2017) 6. doi: 10.7554/eLife.25752 PMC5739539 29266001

[9] Zhang F Zhang H. UDP-glucose ceramide glycosyltransferase contributes to the proliferation and glycolysis of cervical cancer cells by regulating the PI3K/AKT pathway. Ann Clin Lab Sci. (2021) 51:663–9. doi: 0091-7370/21/0500-663 34686508

[10] McKallip R Li R Ladisch S. Tumor gangliosides inhibit the tumor-specific immune response. J Immunol. (1999) 163:3718–26. doi: 10.4049/jimmunol.163.7.3718 10490967

[11] Crocker PR. Siglecs: sialic-acid-binding immunoglobulin-like lectins in cell–cell interactions and signalling. Curr Opin Struct Biol. (2002) 12:609–15. doi: 10.1016/S0959-440X(02)00375-5 12464312

[12] Schauer R. Sialic acids as regulators of molecular and cellular interactions. Curr Opin Struct Biol. (2009) 19:507–14. doi: 10.1016/j.sbi.2009.06.003 PMC7127376 19699080

[13] Seidenfaden R KA Schertzinger F Gerardy-Schahn R Hildebrandt H. Polysialic acid directs tumor cell growth by controlling heterophilic neural cell adhesion molecule interactions. Mol Cell Biol. (2003) 23:5908–18. doi: 10.1128/MCB.23.16.5908-5918.2003 PMC166353 12897159

[14] Toole BP Wight TN Tammi MI. Hyaluronan-cell interactions in cancer and vascular disease. J Biol Chem. (2002) 277:4593–6. doi: 10.1074/jbc.R100039200 11717318

[15] Munkley J Elliott DJ. Hallmarks of glycosylation in cancer. Oncotarget. (2016) 7:35478. doi: 10.18632/oncotarget.v7i23 27007155 PMC5085245

[16] Ghasempour S Freeman SA. The glycocalyx and immune evasion in cancer. FEBS J. (2023) 290:55–65. doi: 10.1111/febs.v290.1 34665926

[17] Offeddu GS Hajal C Foley CR Wan Z Ibrahim L Coughlin MF. The cancer glycocalyx mediates intravascular adhesion and extravasation during metastatic dissemination. Commun Biol. (2021) 4:255. doi: 10.1038/s42003-021-01774-2 33637851 PMC7910477

[18] Gc S Tuy K Rickenbacker L Jones R Chakraborty A Miller CR. [amp]]alpha;2,6 Sialylation mediated by ST6GAL1 promotes glioblastoma growth. JCI Insight. (2022) 7:e158799. doi: 10.1172/jci.insight.158799 36345944 PMC9675560

[19] Krzewinski-Recchi MA JS Juliant S Teintenier-Lelievre M Samyn-Petit B Montiel MD Mir AM. Identification and functional expression of a second human beta-galactoside alpha 2,6-sialyltransferase, ST6Gal II. Eur J Biochem. (2003) 270:950–61. doi: 10.1046/j.1432-1033.2003.03458.x 12603328

[20] Tsuji S Takashima S Angata T. ST6 N-Acetylgalactosaminide Alpha-2,6-Sialyltransferase 2 (ST6GALNAC2). In: Taniguchi N Honke K Fukuda M Narimatsu H Yamaguchi Y, editors. Handbook of Glycosyltransferases and Related Genes. Springer, Tokyo (2014). doi: 10.1007/978-4-431-54240-7_145

[21] Okajima T Furukawa K. ST3 Beta-Galactoside Alpha-2,3-Sialyltransferase 6 (ST3GAL6). In: Taniguchi N Honke K Fukuda M Narimatsu H Yamaguchi Y Angata T, editors. Handbook of Glycosyltransferases and Related Genes. Springer, Tokyo (2014). doi: 10.1007/978-4-431-54240-7_35

[22] Okajima T FS Miyazaki H Ishida H Kiso M Furukawa K Urano T. Molecular cloning of a novel alpha 2,3-sialyltransferase (ST3Gal VI) that sialylates type II lactosamine structures on glycoproteins and glycolipids. J Biol Chem. (1999) 274:11479–86. doi: 10.1074/jbc.274.17.11479 10206952

[23] Yoshida Y Kojima N Kurosawa N Hamamoto T Tsuji S. Molecular cloning of Sia alpha 2,3Gal beta 1,4GlcNAc alpha 2,8-sialyltransferase from mouse brain. J Biol Chem. (1995) 270:14628–33. doi: 10.1074/jbc.270.24.14628 7782326

[24] Takashima S Ishida HK Inazu T Ando T Ishida H Kiso M. Molecular cloning and expression of a sixth type of alpha 2,8-sialyltransferase (ST8Sia VI) that sialylates O-glycans. J Biol Chem. (2002) 277:24030–8. doi: 10.1074/jbc.M112367200 11980897

[25] Yoshida Y Kojima N Tsuji S. Molecular cloning and characterization of a third type of N-glycan alpha 2,8-sialyltransferase from mouse lung. J Biochem. (1995) 118:658–64. doi: 10.1093/oxfordjournals.jbchem.a124960 8690732

[26] Kono M Yoshida Y Kojima N Tsuji S. Molecular cloning and expression of a fifth type of alpha2,8-sialyltransferase (ST8Sia V). Its substrate specificity is similar to that of SAT-V/III, which synthesize GD1c, GT1a, GQ1b and GT3. J Biol Chem. (1996) 271:29366–71. doi: 10.1074/jbc.271.46.29366 8910600

[27] Scott E Archer Goode E Garnham R Hodgson K Orozco-Moreno M Turner H. ST6GAL1-mediated aberrant sialylation promotes prostate cancer progression. J Pathol. (2023) 261:71–84. doi: 10.1002/path.v261.1 37550801

[28] GC S. Understanding the protumorigenic functions of ST6GAL1 in glioblastoma stemness and metabolism. All ETDs from UAB. (2022). Available online at: https://digitalcommons.library.uab.edu/etd-collection/489.

[29] Manhardt CT Punch PR Dougher CW Lau JT. Extrinsic sialylation is dynamically regulated by systemic triggers *in vivo*i. J Biol Chem. (2017) 292:13514–20. doi: 10.1074/jbc.C117.795138 PMC5566511 28717006

[30] Lee MM Nasirikenari M Manhardt CT Ashline DJ Hanneman AJ Reinhold VN. Platelets support extracellular sialylation by supplying the sugar donor substrate. J Biol Chem. (2014) 289:8742–8. doi: 10.1074/jbc.C113.546713 PMC3979374 24550397

[31] PBassagañas S Pérez-Garay M Peracaula R. Cell surface sialic acid modulates extracellular matrix adhesion and migration in pancreatic adenocarcinoma cells. Pancreas. (2014) 43:109–17. doi: 10.1097/MPA.0b013e31829d9090 23921962

[32] Ulrich TA de Juan Pardo EM Kumar S. The mechanical rigidity of the extracellular matrix regulates the structure, motility, and proliferation of glioma cells. Cancer Res. (2009) 69:4167–74. doi: 10.1158/0008-5472.CAN-08-4859 PMC2727355 19435897

[33] Li F Ding J. Sialylation is involved in cell fate decision during development, reprogramming and cancer progression. Protein Cell. (2019) 10:550–65. doi: 10.1007/s13238-018-0597-5 PMC6626595 30478534

[34] Humphrey JD Dufresne ER Schwartz MA. Mechanotransduction and extracellular matrix homeostasis. Nat Rev Mol Cell Biol. (2014) 15:802–12. doi: 10.1038/nrm3896 PMC4513363 25355505

[35] Buffone A Weaver VM. Don’t sugarcoat it: How glycocalyx composition influences cancer progression. J Cell Biol. (2020) 219. doi: 10.1083/jcb.201910070 PMC7039198 31874115

[36] Hudak JE Canham SM Bertozzi CR. Glycocalyx engineering reveals a Siglec-based mechanism for NK cell immunoevasion. Nat Chem Biol. (2014) 10:69–75. doi: 10.1038/nchembio.1388 24292068 PMC3893890

[37] Barkal AA Brewer RE Markovic M Kowarsky M Barkal SA Zaro BW. CD24 signalling through macrophage Siglec-10 is a target for cancer immunotherapy. Nature. (2019) 572:392–6. doi: 10.1038/s41586-019-1456-0 PMC6697206 31367043

[38] Huang Z Guo Y Li B Shen M Yi Y Li L. Siglec-15 on macrophages suppress the immune microenvironment in patients with PD-L1 negative non-metastasis lung adenocarcinoma. Cancer Gene Ther. (2024) 31:427–38. doi: 10.1038/s41417-023-00713-z PMC10940158 38072971

[39] Ibarlucea-Benitez I Weitzenfeld P Smith P Ravetch JV. Siglecs-7/9 function as inhibitory immune checkpoints *in vivo* and can be targeted to enhance therapeutic antitumor immunity. Proc Natl Acad Sci United States America. (2021) 118. doi: 10.1073/pnas.2107424118 PMC8256000 34155121

[40] Zhou X Chi K Zhang C Liu Q Yang G. Sialylation: A cloak for tumors to trick the immune system in the microenvironment. Biology. (2023) 12:832. doi: 10.3390/biology12060832 37372117 PMC10294807

[41] Lee W-L Wang P-H. Aberrant sialylation in ovarian cancers. J Chin Med Assoc. (2020) 83:337–44. doi: 10.1097/JCMA.0000000000000252 PMC13048006 31904658

[42] Rosa-Fernandes L Oba-Shinjo SM Macedo-da-Silva J Marie SKN Palmisano G. Aberrant protein glycosylation in brain cancers, with emphasis on glioblastoma. In: Understanding PTMs in Neurodegenerative Diseases. Springer International Publishing., Cham (2022). p. 39–70. 10.1007/978-3-031-05460-0_4 36029403

[43] Miyagi T. Aberrant expression of sialidase and cancer progression. Proc Jpn Acad Ser B Phys Biol Sci. (2008) 84:407–18. doi: 10.2183/pjab.84.407 PMC3720545 19075514

[44] Munkley J. Aberrant sialylation in cancer: therapeutic opportunities. Cancers. (2022) 14:4248. doi: 10.3390/cancers14174248 36077781 PMC9454432

[45] Munkley J Scott E. Targeting aberrant sialylation to treat cancer. Medicines. (2019) 6:102. doi: 10.3390/medicines6040102 31614918 PMC6963943

[46] Orozco-Moreno M Visser EA Hodgson K Hipgrave Ederveen AL Bastian K Goode EA. Targeting aberrant sialylation and fucosylation in prostate cancer cells using potent metabolic inhibitors. Glycobiology. (2023) 33:1155–71. doi: 10.1093/glycob/cwad085 PMC10876042 37847613

[47] Chen X Varki A. Advances in the biology and chemistry of sialic acids. ACS Chem Biol. (2010) 5:163–76. doi: 10.1021/cb900266r PMC2825284 20020717

[48] Li Y Chen X. Sialic acid metabolism and sialyltransferases: natural functions and applications. Appl Microbiol Biotechnol. (2012) 94:887–905. doi: 10.1007/s00253-012-4040-1 22526796 PMC3534974

[49] Angata T Varki A. Chemical diversity in the sialic acids and related α-keto acids: an evolutionary perspective. Chem Rev. (2002) 102:439–70. doi: 10.1021/cr000407m 11841250

[50] Schauer R. Achievements and challenges of sialic acid research. Glycoconjugate J. (2000) 17:485–99. doi: 10.1023/A:1011062223612 PMC7087979 11421344

[51] Varki A Cummings RD Esko JD Stanley P Hart GW Aebi M. Essentials of Glycobiology. 2nd edn Vol. 2008. United States: Cold Spring Harbor Laboratory (2015) p. 199–217. 27010055

[52] Varki A Cummings RD Esko JD Freeze HH Stanley P Bertozzi CR eds. Essentials of glycobiology. 2nd edn. Cold Spring Harbor: Cold Spring Harbor Laboratory (2008) p. 199–217. 20301239

[53] Yu H Zeng J Li Y Thon V Shi B Chen X. Effective one-pot multienzyme (OPME) synthesis of monotreme milk oligosaccharides and other sialosides containing 4-O-acetyl sialic acid. Organic biomolecular Chem. (2016) 14:8586–97. doi: 10.1039/C6OB01706A PMC5036589 27548611

[54] Visser EA Moons SJ Timmermans SB de Jong H Boltje TJ Büll C. Sialic acid O-acetylation: From biosynthesis to roles in health and disease. J Biol Chem. (2021) 297. doi: 10.1016/j.jbc.2021.100906 PMC8319020 34157283

[55] Park SS. Post-glycosylation modification of sialic acid and its role in virus pathogenesis. Vaccines. (2019) 7:171. doi: 10.3390/vaccines7040171 31683930 PMC6963189

[56] Toba S Tenno M Kurosaka A. An O-acetylated sialyl-Tn is involved in ovarian cancer-associated antigenicity. Biochem Biophys Res Commun. (2000) 271:281–6. doi: 10.1006/bbrc.2000.2618 10799288

[57] Ariga T Blaine GM Yoshino H. Glycosphingolipid composition of murine neuroblastoma cells: O-acetylesterase gene downregulates the expression of O-acetylated GD*3* . Biochemistry. (1995) 34:11500–7. doi: 10.1021/bi00036a024 7547879

[58] Sinha D Mandal C Bhattacharya DK. Identification of 9-O acetyl sialoglycoconjugates (9-OAcSGs) as biomarkers in childhood acute lymphoblastic leukemia using a lectin, AchatininH, as a probe. Leukemia. (1999) 13:119–25. doi: 10.1038/sj.leu.2401239 PMC7100560 10049046

[59] Mukherjee K Chava AK Mandal C. O-acetylation of GD3 prevents its apoptotic effect and promotes survival of lymphoblasts in childhood acute lymphoblastic leukaemia. J Cell Biochem. (2008) 105:724–34. doi: 10.1002/jcb.v105:3 18655184

[60] Mather RL Loveson KF Fillmore. HL. Human Sialic acid O-acetyl esterase (SIAE) - mediated changes in sensitivity to etoposide in a medulloblastoma cell line. Sci Rep. (2019) 9:8609. doi: 10.1038/s41598-019-44950-5 31197190 PMC6565703

[61] Marquina G Waki H Fernandez LE. Gangliosides expressed in human breast cancer. Cancer Res. (1996) 56:5165–71. 8912852

[62] Zhang Z Wuhrer M Holst S. Serum sialylation changes in cancer. Glycoconjugate J. (2018) 35:139–60. doi: 10.1007/s10719-018-9820-0 PMC5916985 29680984

[63] Sun H Zhou Y Jiang H Xu Y. Elucidation of functional roles of sialic acids in cancer migration. Front Oncol. (2020) 10:401. doi: 10.3389/fonc.2020.00401 32296639 PMC7137995

[64] Schwedler C Kaup M Petzold D Hoppe B Braicu EI Sehouli J. Sialic acid methylation refines capillary electrophoresis laser-induced fluorescence analyses of immunoglobulin GN-glycans of ovarian cancer patients. Electrophoresis. (2014) 35:1025–31. doi: 10.1002/elps.201300414 24812685

[65] Dobie C Skropeta D. Insights into the role of sialylation in cancer progression and metastasis. Br J Cancer. (2021) 124:76–90. doi: 10.1038/s41416-020-01126-7 33144696 PMC7782833

[66] Skropeta D. The effect of individual N-glycans on enzyme activity. Bioorganic medicinal Chem. (2009) 17:2645–53. doi: 10.1016/j.bmc.2009.02.037 19285412

[67] Varki A. Sialic acids in human health and disease. Trends Mol Med. (2008) 14:351–60. doi: 10.1016/j.molmed.2008.06.002 PMC2553044 18606570

[68] Lairson LL Henrissat B Davies GJ Withers SG. Glycosyltransferases: structures, functions, and mechanisms. Annu Rev Biochem. (2008) 77:521–55. doi: 10.1146/annurev.biochem.76.061005.092322 18518825

[69] Hinderlich S Stasche R Zeitler R Reutter W. A bifunctional enzyme catalyzes the first two steps in N-acetylneuraminic acid biosynthesis of rat liver. Purification and characterization of UDP-N-acetylglucosamine 2-epimerase/N-acetylmannosamine kinase. J J Biol Chem. (1997) 272:24313–8. doi: 10.1074/jbc.272.39.24313 9305887

[70] Roseman S JG Watson D Rood R. Enzymatic synthesis of sialic acid 9-phosphates. Proc Natl Acad Sci USA. (1961) 47:958–61. doi: 10.1073/pnas.47.7.958 PMC221309 13743311

[71] Roseman S Comb DG Ginsburg V Huennekens FM. Enzymatic synthesis of sialic acid 9-phosphate and N-acetylneuraminic acid. J Biol Chem. (1961) 236:1945–50. doi: 10.1073/pnas.47.7.958

[72] Willems AP Sun L Schulz MA Tian W Ashikov A van Scherpenzeel M. Activity of N-acylneuraminate-9-phosphatase (NANP) is not essential for de novo sialic acid biosynthesis. Biochim Biophys Acta Gen Subj. (2019) 1863(10):1471–9. doi: 10.1016/j.bbagen.2019.05.011 31121216

[73] Kean EL Roseman S. The synthesis of cytidine 5′-monophospho-N-acetylneuraminic acid by a nuclear membrane fraction from eel hepatopancreas. J Biol Chem. (1966) 241:5642–50. doi: 10.2147/OTT.S177639

[74] Kitagawa H Paulson JC. Cloning and expression of human Gal beta 1,3(4)GlcNAc alpha 2,3-sialyltransferase. J Biol Chem. (1994) 269:5665–71. doi: 10.1006/bbrc.1993.1830 8333853

[75] Kim YJ Kim KS Kim SH Kim CH Ko JH Choe IS. Molecular cloning and expression of human Galβ1, 3GalNAc α2, 3-sialyltransferase (hST3Gal II). Biochem Biophys Res Commun. (1996) 228(2):324–7. doi: 10.1006/bbrc.1996.1660 8920913

[76] Tsuji S Datta AK Paulson JC. Systematic nomenclature for sialyltransferases. Glycobiology. (1996) 6:613–24. doi: 10.1093/glycob/6.7.647-d 8953271

[77] Lee YC KN Wada E Kurosawa N Nakaoka T Hamamoto T Tsuji S. Cloning and expression of cDNA for a new type of Gal beta 1,3GalNAc alpha 2,3-sialyltransferase. J Biol Chem. (1994) 269:10028–33. doi: 10.1016/S0021-9258(17)36985-5 8144500

[78] Okajima M Fukumoto S Miyazaki H Hasegawa T Furukawa K Furukawa K. Molecular cloning and functional analysis of human ST3Gal IV, a novel member of the Galβ1,3(4)GlcNAc α2,3-sialyltransferase family. J Biol Chem. (1999) 274:11479–84. doi: 10.1074/jbc.274.17.11479 10206952

[79] Nara K Watanabe Y Maruyama K Kasahara K Nagai Y Sanai Y. Expression cloning of a CMP-NeuAc:NeuAc alpha 2-3Gal beta 1-4Glc beta 1-1'Cer alpha 2,8-sialyltransferase (GD3 synthase) from human melanoma cells. Proc Natl Acad Sci USA. (1994) 91(17):7952–6. doi: 10.1073/pnas.91.17.7952 PMC44522 8058740

[80] Kitagawa H Paulson JC. Cloning and expression of human Galβ1, 3 (4) GlcNAc α2, 3-sialyltransferase. Biochem Biophys Res Commun. (1993) 194(1):375–82. doi: 10.1006/bbrc.1993.1830 8333853

[81] Takashima S Tsuji S Tsujimoto M. Characterization of the second type of human β-galactoside α2, 6-sialyltransferase (ST6Gal II), which sialylates Galβ1, 4GlcNAc structures on oligosaccharides preferentially: Genomic analysis of human sialyltransferase genes. J Biol Chem. (2002) 277:45719–28. doi: 10.1074/jbc.M206808200 12235148

[82] Kurosawa N Takashima S Kono M Ikehara Y Inoue M Tachida Y. Molecular cloning and genomic analysis of mouse GalNAc alpha2, 6-sialyltransferase (ST6GalNAc I). J Biochem. (2000) 127(5):845–54. doi: 10.1093/oxfordjournals.jbchem.a022678 10788794

[83] Takashima S TY Nakagawa T Hamamoto T Tsuji S. Quantitative analysis of expression of mouse sialyltransferase genes by competitive PCR. Biochem Biophys Res Commun. (1999) 260:23–7. doi: 10.1006/bbrc.1999.0794 10381338

[84] Lee YC Kaufmann M Kitazume-Kawaguchi S Kono M Takashima S Kurosawa N. Molecular cloning and functional expression of two members of mouse NeuAcalpha2,3Galbeta1,3GalNAc GalNAcalpha2,6-sialyltransferase family, ST6GalNAc III and IV. J Biol Chem. (1999) 274:11958–67. doi: 10.1074/jbc.274.17.11958 10207017

[85] Harduin-Lepers A Stokes DC Steelant WF Samyn-Petit B Krzewinski-Recchi MA Vallejo-Ruiz V. Cloning, expression and gene organization of a human Neu5Acα2–3Galβ1–3GalNAc α2, 6-sialyltransferase: hST6GalNAc IV. Biochem J. (2000) 352:37–48. doi: 10.1042/bj3520037 11062056 PMC1221430

[86] Ikehara Y SN Kono M Nishihara S Nakanishi H Kitamura T Narimatsu H. A novel glycosyltransferase with a polyglutamine repeat; a new candidate for GD1alpha synthase (ST6GalNAc V)(1). FEBS Lett. (1999) 463:92–6. doi: 10.1016/S0014-5793(99)01605-1 10601645

[87] Okajima T CH Ito H Kiso M Tai T Furukawa K Urano T. Molecular cloning and expression of mouse GD1alpha/GT1aalpha/GQ1balpha synthase (ST6GalNAc VI) gene. Biol Chem. (2000) 275:6717–23. doi: 10.1074/jbc.275.10.6717 10702226

[88] Delannoy P. Role of complex gangliosides in cancer progression. In: Carbohydrate chemistry. United Kingdom: Taylor and francis. (2011) 37:1–20.

[89] Eckhardt M G-SR. Genomic organization of the murine polysialyltransferase gene ST8SiaIV (PST-1). Glycobiology. (1998) 8:1165–72. doi: 10.1093/glycob/8.12.1165 9858638

[90] Isaji T Im S Kameyama A Wang Y Fukuda T Gu J. A complex between phosphatidylinositol 4-kinase IIα and integrin α3β1 is required for N-glycan sialylation in cancer cells. J Biol Chem. (2019) 294:4425–36. doi: 10.1074/jbc.RA118.005208 PMC6433072 30659093

[91] Munkley J Oltean S Vodák D Wilson BT Livermore KE Zhou Y. The androgen receptor controls expression of the cancer-associated sTn antigen and cell adhesion through induction of ST6GalNAc1 in prostate cancer. Oncotarget. (2015) 6:34358–74. doi: 10.18632/oncotarget.v6i33 PMC4741458 26452038

[92] Akamine S NT Sawai T Tsuji T Tanaka K Hidaka S. Differences in prognosis of col-orectal cancer patients based on the expression ofsialyl Lewisa, sialyl Lewisx and sialyl Tn antigensin serum and tumor tissue. Anticancer Res. (2004) 24:2541–6, 2004. 15330211

[93] Dougher CWL Buffone A Jr. Nemeth MJ Nasirikenari M Irons EE Bogner PN. The blood-borne sialyltransferase ST6Gal-1 is a negative systemic regulator of granulopoiesis. J Leukoc Biol. (2017) 102:507–16. doi: 10.1189/jlb.3A1216-538RR PMC5505748 28550122

[94] Nakamori S Kameyama M Imaoka S Furukawa H Ishikawa O Sasaki Y. Increased expression of sialyl Lewisx antigen correlates with poor survival in patients with colorectal carcinoma: clinicopathological and immunohistochemical study. Cancer Res. (1993) 53:3632–7. Available online at: https://aacrjournals.org/cancerres/article/53/15/3632/499029/Increased-Expression-of-Sialyl-Lewisx-Antigen. 8101764

[95] Amado M Carneiro F Seixas M Clausen H Sobrinho–Simões M. Dimeric sialyl-Lex expression in gastric carcinoma correlates with venous invasion and poor outcome. Gastroenterology. (1998) 114:462–70. doi: 10.1016/S0016-5085(98)70529-3 9496936

[96] Liu YC Yen HY Chen CY Chen CH Cheng PF Juan YH. Sialylation and fucosylation of epidermal growth factor receptor suppress its dimerization and activation in lung cancer cells. Proc Natl Acad Sci. (2011) 108:11332–7. doi: 10.1073/pnas.1107385108 PMC3136320 21709263

[97] Büll C Boltje TJ Balneger N Weischer SM Wassink M van Gemst JJ. Sialic acid blockade suppresses tumor growth by enhancing T-cellMediated tumor immunity. Cancer Res. (2018) 78:3574–88. doi: 10.1158/0008-5472.CAN-17-3376 29703719

[98] Schwarzkopf M KK Rohde E Hinderlich S Wiechens N Lucka L Horak I. Sialylation is essential for early development in mice. Proc Natl Acad Sci USA. (2002) 99:5267–70. doi: 10.1073/pnas.072066199 PMC122758 11929971

[99] Park S Colville MJ Paek JH Shurer CR Singh A Secor EJ. Immunoengineering can overcome the glycocalyx armour of cancer cells. Nat Materials. (2024), 1–10. doi: 10.1038/s41563-024-01808-0 38361041 PMC11471287

[100] Yardeni T CT Jacobs K Ciccone C Patzel K Anikster Y Gahl WA. Identification, tissue distribution, and molecular modeling of novel human isoforms of the key enzyme in sialic acid synthesis, UDP-GlcNAc 2-epimerase/ManNAc kinase. Biochemistry. (2011) 50:8914–25. doi: 10.1021/bi201050u PMC3192532 21910480

[101] Verheijen FW VE Aula N Beerens CE Havelaar AC Joosse M Peltonen L. A new gene, encoding an anion transporter, is mutated in sialic acid storage diseases. Nat Genet. (1999) 23:462–5. doi: 10.1038/70585 10581036

[102] Pshezhetsky AV RC Michaud L Igdoura S Wang S Elsliger MA Qu J. Cloning, expression and chromosomal mapping of human lysosomal sialidase and characterization of mutations in sialidosis. Nat Genet. (1997) 15:316–20. doi: 10.1038/ng0397-316 9054950

[103] Bonten E van der Spoel A Fornerod M Grosveld G d’Azzo A. Characterization of human lysosomal neuraminidase defines the molecular basis of the metabolic storage disorder sialidosis. Genes Dev. (1996) 10:3156–69. doi: 10.1101/gad.10.24.3156 8985184

[104] Monti E PA Rossi E Ballabio A Borsani G. Cloning and characterization of NEU2, a human gene homologous to rodent soluble sialidases. Genomics. (1999) 57:137–43. doi: 10.1006/geno.1999.5749 10191093

[105] Wada T YY Tokuyama S Kuwabara M Akita H Miyagi T. Cloning, expression, and chromosomal mapping of a human ganglioside sialidase. Biochem Biophys Res Commun. (1999) 261:21–27 1999. doi: 10.1006/bbrc.1999.0973 10405317

[106] Monti E BM Bresciani R Civini S Croci GL Papini N Riboni M. Molecular cloning and characterization of NEU4, the fourth member of the human sialidase gene family. Genomics. (2004) 83:445–53. doi: 10.1016/j.ygeno.2003.08.019 14962670

[107] Miyagi T WT Yamaguchi K Hata K. Sialidase and Malignancy: a minireview. Glycoconj J. (2004) 20:189–98. doi: 10.1023/B:GLYC.0000024250.48506.bf 15090732

[108] Irons EE Gc S Lau JTY. Sialic acid in the regulation of blood cell production, differentiation and turnover. Immunology. (2024) 172:517–32. doi: 10.1111/imm.v172.4 PMC11223974 38503445

[109] Hait NC Maiti A Wu R Andersen VL Hsu CC Wu Y. Extracellular sialyltransferase st6gal1 in breast tumor cell growth and invasiveness. Cancer Gene Ther. (2022) 29:1662–75. doi: 10.1038/s41417-022-00485-y PMC9663294 35676533

[110] Varki A Cummings RD Esko JD Stanley P Hart GW Aebi M. Chapter 17 Glycosyltransferases, in Essentials of Glycobiology. Cold Spring Harbor Laboratory Press Copyright 2015-2017 by The Consortium of Glycobiology Editors, La Jolla, California, Cold Spring Harbor (NY (2015).

[111] Wandall HH Rumjantseva V Sørensen AL Patel-Hett S Josefsson EC Bennett EP. The origin and function of platelet glycosyltransferases. Blood. (2012) 120:626–35. doi: 10.1182/blood-2012-02-409235 PMC3401214 22613794

[112] Lee-Sundlov MM Ashline DJ Hanneman AJ Grozovsky R Reinhold VN Hoffmeister KM. Circulating blood and platelets supply glycosyltransferases that enable extrinsic extracellular glycosylation. Glycobiology. (2016). doi: 10.1093/glycob/cww108 PMC5224594 27798070

[113] Nasirikenari M Veillon L Collins CC Azadi P Lau JTY. Remodeling of marrow hematopoietic stem and progenitor cells by non-self ST6Gal-1 sialyltransferase. J Biol Chem. (2014) 289:7178–89. doi: 10.1074/jbc.M113.508457 PMC3945377 24425878

[114] Rusiniak ME Punch PR Hait NC Maiti A Burns RT Chapla D. Extracellular ST6GAL1 regulates monocyte-macrophage development and survival. Glycobiology. (2022) 32:701–11. doi: 10.1093/glycob/cwac032 PMC9280526 35661210

[115] Irons EE Lau JTY. Systemic ST6Gal-1 is a pro-survival factor for murine transitional B cells. Front Immunol. (2150) 2018:9. doi: 10.3389/fimmu.2018.02150 PMC6159744 30294329

[116] Irons EE Lee-Sundlov MM Zhu Y Neelamegham S Hoffmeister KM Lau JTY. B cells suppress medullary granulopoiesis by an extracellular glycosylation-dependent mechanism. eLife. (2019) 8:e47328. doi: 10.7554/eLife.47328.014 31408003 PMC6713473

[117] Irons EE Punch PR Lau JTY. Blood-borne ST6GAL1 regulates immunoglobulin production in B cells. Front Immunol. (2020) 11:617. doi: 10.3389/fimmu.2020.00617 32391003 PMC7190976

[118] Punch PR Irons EE Manhardt CT Marathe H Lau JTY. The sialyltransferase ST6GAL1 protects against radiation-induced gastrointestinal damage. Glycobiology. (2020) 30:446–53. doi: 10.1093/glycob/cwz108 PMC7305799 31897489

[119] Glendenning LM Zhou JY Reynero KM Cobb BA. Divergent Golgi trafficking limits B cell-mediated IgG sialylation. J leukocyte Biol. (2022) 112:1555–66. doi: 10.1002/JLB.3MA0522-731R PMC9701147 35726710

[120] Jones MB Oswald DM Joshi S Whiteheart SW Orlando R Cobb BA. B-cell-independent sialylation of igG. Proc Natl Acad Sci United States America. (2016) 113:7207–12. doi: 10.1073/pnas.1523968113 PMC4932940 27303031

[121] Wang J Shewell LK Day CJ Jennings MP. N-glycolylneuraminic acid as a carbohydrate cancer biomarker. Trans Oncol. (2023) 31:101643. doi: 10.1016/j.tranon.2023.101643 PMC9971276 36805917

[122] Padler-Karavani V Yu H Cao H Chokhawala H Karp F Varki N. Diversity in specificity, abundance, and composition of anti-Neu5Gc antibodies in normal humans: potential implications for disease. Glycobiology. (2008) 18:818–30. doi: 10.1093/glycob/cwn072 PMC2586336 18669916

[123] Samraj AN Läubli H Varki N Varki A. Involvement of a non-human sialic acid in human cancer. Front Oncol. (2014) 4:33. doi: 10.3389/fonc.2014.00033 24600589 PMC3928833

[124] Malykh YN Schauer R Shaw L. N-Glycolylneuraminic acid in human tumours. Biochimie. (2001) 83:623–34. doi: 10.1016/S0300-9084(01)01303-7 11522391

[125] Samraj AN Pearce OM Läubli H Crittenden AN Bergfeld AK Banda K. A red meat-derived glycan promotes inflammation and cancer progression. Proc Natl Acad Sci. (2015) 112:542–7. doi: 10.1073/pnas.1417508112 PMC4299224 25548184

[126] Tangvoranuntakul P Gagneux P Diaz S Bardor M Varki N Varki A. Human uptake and incorporation of an immunogenic nonhuman dietary sialic acid. Proc Natl Acad Sci. (2003) 100:12045–50. doi: 10.1073/pnas.2131556100 PMC218710 14523234

[127] Altman MO Gagneux P. Absence of Neu5Gc and presence of anti-Neu5Gc antibodies in humans—an evolutionary perspective. Front Immunol. (2019) 10:789. doi: 10.3389/fimmu.2019.00789 31134048 PMC6524697

[128] Dhar C Sasmal A Varki A. From “serum sickness” to “xenosialitis”: past, present, and future significance of the non-human sialic acid Neu5Gc. Front Immunol. (2019) 10:807. doi: 10.3389/fimmu.2019.00807 31057542 PMC6481270

[129] Afrasiabi K Zhou YH Fleischman A. Chronic inflammation: is it the driver or is it paving the road for Malignant transformation? Genes Cancer. (2015) 6:214. doi: 10.18632/genesandcancer.64 26124920 PMC4482242

[130] Zhao H Wu L Yan G Chen Y Zhou M Wu Y. Inflammation and tumor progression: signaling pathways and targeted intervention. Signal transduction and targeted therapy. Signal Trans Ther (2021) 6(1):263. doi: 10.1038/s41392-021-00658-5 PMC8273155 34248142

[131] Ghosh S. Sialic acids and sialoglycans in endocrinal disorders. Sialic Acids and Sialoglycoconjugates in the Biology of Life, Health and Disease. United States: Academic Press (2020) p. 247–68. doi: 10.1016/B978-0-12-816126-5.00009-3

[132] Dall'Olio F Chiricolo M. Sialyltransferases in cancer. Glycoconjugate J. (2001) 18:841–50. doi: 10.1023/A:1022288022969 12820717

[133] Pinho SaloméS Reis CA. Glycosylation in cancer: mechanisms and clinical implications. Nat Rev Cancer. (2015) 15:540–55. doi: 10.1038/nrc3982 26289314

[134] Saeui CT Cho KC Dharmarha V Nairn AV Galizzi M Shah SR. Cell line-, protein-, and sialoglycosite-specific control of flux-based sialylation in human breast cells: implications for cancer progression. Front Chem. (2020) 8:13. doi: 10.3389/fchem.2020.00013 32117864 PMC7013041

[135] Scott E Elliott DJ Munkley J. Tumour associated glycans: A route to boost immunotherapy? Clinica Chimica Acta. (2020) 502:167–73. doi: 10.1016/j.cca.2019.12.015 31870793

[136] Garnham R Scott E Livermore KE Munkley J. ST6GAL1: A key player in cancer. Oncol Lett. (2019) 18:983–9. doi: 10.3892/ol.2019.10458 PMC6607188 31423157

[137] Ghosh S Bandyopadhyay S Mukherjee K Mallick A Pal S Mandal C. O-acetylation of sialic acids is required for the survival of lymphoblasts in childhood acute lymphoblastic leukemia (ALL). Glycoconjugate J. (2007) 24:17–24. doi: 10.1007/s10719-006-9007-y 17146715

[138] Hauselmann I Borsig L. Altered tumor-cell glycosylation promotes metastasis. Front Oncol. (2014) 4:28. doi: 10.3389/fonc.2014.00028 24592356 PMC3923139

[139] Piplani N Roy T Saxena N Sen S. Bulky glycocalyx shields cancer cells from invasion-associated stresses. Trans Oncol. (2024) 39:101822. doi: 10.1016/j.tranon.2023.101822 PMC10654248 37931370

[140] Luo Y Cao H Lei C Liu J. ST6GALNAC1 promotes the invasion and migration of breast cancer cells via the EMT pathway. Genes Genomics. (2023) 45:1367–76. doi: 10.1007/s13258-023-01445-y 37747641

[141] Ma X Dong W Su Z Zhao L Miao Y Li N. Functional roles of sialylation in breast cancer progression through miR-26a/26b targeting ST8SIA4. Cell Death Dis. (2016) 7:e2561–1. doi: 10.1038/cddis.2016.427 PMC5260976 28032858

[142] Hodgson K Orozco-Moreno M Goode EA Fisher M Garnham R Beatson R. Sialic acid blockade inhibits the metastatic spread of prostate cancer to bone. EBioMedicine. (2024) 104. doi: 10.1016/j.ebiom.2024.105163 PMC11134892 38772281

[143] Takeyama H Kyoda S Okamoto T Manome Y Watanabe M Kinoshita S. The expression of sialic fibronectin correlates with lymph node metastasis of thyroid Malignant neoplasmas. Anticancer Res. (2011) 31:1395–8. 21508391

[144] Vierbuchen M Schröder S Larena A Uhlenbruck G Fischer R. Native and sialic acid masked Lewis(a) antigen reactivity in medullary thyroid carcinoma. Distinct tumour-associated and prognostic relevant antigens. Virchows Arch. (1994) 424:205–11. doi: 10.1007/BF00193501 8180782

[145] Jiang MS Passaniti A Penno MB Hart GW. Adrenal carcinoma tumor progression and penultimate cell surface oligosaccharides. Cancer Res. (1992) 52:2222–7. 1559226

[146] Dwivedi C Dixit M Hardy RE. Plasma lipid-bound sialic acid alterations in neoplastic diseases. Experientia. (1990) 46:91–4. doi: 10.1007/BF01955427 2298288

[147] Ozyurt E Sönmez H Süer S Kökoğlu E. The prognostic importance of fibronectin and sialic acid levels in human pituitary adenomas. Cancer Lett. (1996) 100:151–4. doi: 10.1016/0304-3835(95)04086-2 8620435

[148] Kökoğlu E Süer S Ozyurt E Siyahhan A Sönmez H. Plasma fibronectin and sialic acid levels in various types of human brain tumors. Cancer Biochem Biophys. (1995) 15:35–40. 8536218

[149] Trouillas J Daniel L Guigard MP Tong S Gouvernet J Jouanneau E. Polysialylated neural cell adhesion molecules expressed in human pituitary tumors and related to extrasellar invasion. J Neurosurg. (2003) 98:1084–93. doi: 10.3171/jns.2003.98.5.1084 12744370

[150] Sakuma K Akiyama Y Sakamoto M Yamamoto Y Uchimura K Nishimura SI. Role of ST3GAL1, ST3GAL3, and ST3GAL4 in the regulation of cancer metastasis through sialylation of glycoproteins and glycolipids. Glycobiology. (2012) 22:877–87. doi: 10.1093/glycob/cws071

[151] Fan TC Yeo HL Hsu HM Yu JC Ho MY Lin WD. Reciprocal feedback regulation of ST3GAL1 and GFRA1 signaling in breast cancer cells. Cancer Lett. (2018) 434:184–95. doi: 10.1016/j.canlet.2018.07.026 30040982

[152] Zhang SZ Lobo A Li PF Zhang YF. Sialylated glycoproteins and sialyltransferases in digestive cancers: mechanisms, diagnostic biomarkers, and therapeutic targets. Crit Rev Oncology/Hematol. (2024), 104330. doi: 10.1016/j.critrevonc.2024.104330 38556071

[153] Wu X Zhao J Ruan Y Sun L Xu C Jiang H. Sialyltransferase ST3GAL1 promotes cell migration, invasion, and TGF-β1-induced EMT and confers paclitaxel resistance in ovarian cancer. Cell Death Dis. (2018) 9:1102. doi: 10.1038/s41419-018-1101-0 30375371 PMC6207573

[154] Yamamoto H Oviedo A Sweeley C Saito T Moskal JR. A2, 6-sialylation of cell-surface N-glycans inhibits glioma formation *in vivo* . Cancer Res. (2001) 61:6822–9. 11559557

[155] Wen KC Sung PL Hsieh SL Chou YT Lee OKS Wu CW. [amp]]alpha;2, 3-sialyltransferase type I regulates migration and peritoneal dissemination of ovarian cancer cells. Oncotarget. (2017) 8:29013. doi: 10.18632/oncotarget.15994 28423672 PMC5438708

[156] Shen L Luo Z Wu J Qiu L Luo M Ke Q. Enhanced expression of α2, 3-linked sialic acids promotes gastric cancer cell metastasis and correlates with poor prognosis. Int J Oncol. (2017) 50:1201–10. doi: 10.3892/ijo.2017.3882 28259967

[157] Mondal S Chandra S Mandal C. Elevated mRNA level of hST6Gal I and hST3Gal V positively correlates with the high risk of pediatric acute leukemia. Leukemia Res. (2010) 34:463–70. doi: 10.1016/j.leukres.2009.07.042 19709745

[158] Swindall AF Bellis SL. Sialylation of the Fas death receptor by ST6Gal-I provides protection against Fas-mediated apoptosis in colon carcinoma cells. J Biol Chem. (2011) 286:22982–90. doi: 10.1074/jbc.M110.211375 PMC3123066 21550977

[159] Rao TC Beggs RR Ankenbauer KE Hwang J Ma VPY Salaita K. ST6Gal-I–mediated sialylation of the epidermal growth factor receptor modulates cell mechanics and enhances invasion. J Biol Chem. (2022) 298. doi: 10.1016/j.jbc.2022.101726 PMC8956946 35157848

[160] Venturi G. The impact of ST6Gal-I in the progression of colorectal cancer. Alma Mater Studiorum Università di Bologna. Dottorato di ricerca in Oncologia, ematologia e patologia, 30 Ciclo, Bologna-Italy (2018). doi: 10.6092/unibo/amsdottorato/8663

[161] Dorsett KA. Regulation of ST6Gal-I in cancer: SOX2 identified as novel driver of ST6Gal-I expression. The University of Alabama at Birmingham, Birmingham AL USA (2019).

[162] Britain CM. Receptor sialylation by ST6Gal-I promotes tumor progression by enhancing tumor cell survival and epithelial to mesenchymal transition. The University of Alabama at Birmingham, Birmingham AL, United States (2019).

[163] Jung YuRi Park J-J Jin YB Cao YJ Park M-J Kim EJu. Silencing of ST6Gal I enhances colorectal cancer metastasis by down-regulating KAI1 via exosome-mediated exportation and thereby rescues integrin signaling. Carcinogenesis. (2016) 37:1089–97. doi: 10.1093/carcin/bgw091 27559112

[164] Cheng J Wang R Zhong G Chen X Cheng Y Li W. RETRACTED ARTICLE: ST6GAL2 downregulation inhibits cell adhesion and invasion and is associated with improved patient survival in breast cancer. OncoTargets Ther. (2020) 2020:903–14. doi: 10.2147/OTT.S230847 PMC6996233 32099394

[165] Yu X Wu Q Wang L Zhao Y Zhang Q Meng Q. Silencing of ST6GalNAc I suppresses the proliferation, migration and invasion of hepatocarcinoma cells through PI3K/AKT/NF-κB pathway. Tumor Biol. (2016) 37:12213–21. doi: 10.1007/s13277-016-5086-y 27235117

[166] Ren D Jia L Li Y Gong Y Liu C Zhang X. ST6GalNAcII mediates the invasive properties of breast carcinoma through PI3K/Akt/NF-κB signaling pathway. IUBMB Life. (2014) 66:300–8. doi: 10.1002/iub.v66.4 24756995

[167] Man D Jiang Y Zhang D Wu J Ding B Liu H. ST6GALNAC4 promotes hepatocellular carcinogenesis by inducing abnormal glycosylation. J Trans Med. (2023) 21:420. doi: 10.1186/s12967-023-04191-7 PMC10308692 37381011

[168] Dai T Li J Liang RB Yu H Lu X Wang G. Identification and experimental validation of the prognostic significance and immunological correlation of glycosylation-related signature and ST6GALNAC4 in hepatocellular carcinoma. J Hepatocellular Carcinoma. (2023), 531–51. doi: 10.2147/JHC.S400472 PMC10081533 37034303

[169] Li M Ma Z Zhang Y Feng H Li Y Sang W. Integrative analysis of the ST6GALNAC family identifies GATA2-upregulated ST6GALNAC5 as an adverse prognostic biomarker promoting prostate cancer cell invasion. Cancer Cell Int. (2023) 23:141. doi: 10.1186/s12935-023-02983-x 37468844 PMC10355028

[170] Kroes RA He H Emmett MR Nilsson CL Leach FE III Amster IJ. Overexpression of ST6GalNAcV, a ganglioside-specific α2, 6-sialyltransferase, inhibits glioma growth *in vivo* . Proc Natl Acad Sci. (2010) 107:12646–51. doi: 10.1073/pnas.0909862107 PMC2906591 20616019

[171] Yan Y Nguyen K Do KA Ueno N Andreeff M Battula VL. Abstract P6-07-26: ST8SIA1 is over-expressed in triple negative breast cancer and associated with p53 mutations. Cancer Res. (2017) 77:P6–07. doi: 10.1158/1538-7445.SABCS16-P6-07-26

[172] Ramos RI Bustos MA Wu J Jones P Chang SC Kiyohara E. Upregulation of cell surface GD3 ganglioside phenotype is associated with human melanoma brain metastasis. Mol Oncol. (2020) 14:1760–78. doi: 10.1002/1878-0261.12702 PMC7400791 32358995

[173] Wang N Cao S Wang X Zhang L Yuan H Ma X. lncRNA MALAT1/miR−26a/26b/ST8SIA4 axis mediates cell invasion and migration in breast cancer cell lines. Oncol Rep. (2021) 46:1–12. doi: 10.3892/or.2021.8132 PMC8273684 34278507

[174] Ma W Zhao X Liang L Wang G Li Y Miao X. miR-146a and miR-146b promote proliferation, migration and invasion of follicular thyroid carcinoma via inhibition of ST8SIA4. Oncotarget. (2017) 8:28028. doi: 10.18632/oncotarget.15885 28427206 PMC5438628

[175] Fei Q Song F Jiang X Hong H Xu X Jin Z. LncRNA ST8SIA6-AS1 promotes hepatocellular carcinoma cell proliferation and resistance to apoptosis by targeting miR-4656/HDAC11 axis. Cancer Cell Int. (2020) 20:1–10. doi: 10.1186/s12935-020-01325-5 32536820 PMC7288512

[176] Fang K Hu C Zhang X Hou Y Gao D Guo Z. LncRNA ST8SIA6-AS1 promotes proliferation, migration and invasion in breast cancer through the p38 MAPK signalling pathway. Carcinogenesis. (2020) 41:1273–81. doi: 10.1093/carcin/bgz197 31784750

[177] Cao Q Yang W Ji X Wang W. Long non-coding RNA ST8SIA6-AS1 promotes lung adenocarcinoma progression through sponging miR-125a-3p. Front Genet. (2020) 11:597795. doi: 10.3389/fgene.2020.597795 33363573 PMC7753099

[178] Kufe DW. Mucins in cancer: function, prognosis and therapy. Nat Rev Cancer. (2009) 9:874–85. doi: 10.1038/nrc2761 PMC2951677 19935676

[179] Horm TM Schroeder JA. MUC1 and metastatic cancer: expression, function and therapeutic targeting. Cell Adh Migr. (2013) 7:187–98. doi: 10.4161/cam.23131 PMC3954031 23303343

[180] Hollingsworth MA Swanson BJ. Mucins in cancer: protection and control of the cell surface. Nat Rev Cancer. (2004) 4:45–60. doi: 10.1038/nrc1251 14681689

[181] Macauley MS Paulson JC. Siglecs induce tolerance to cell surface antigens by BIM-dependent deletion of the antigen-reactive B cells. J Immunol. (2014) 193:4312–21. doi: 10.4049/jimmunol.1401723 PMC4201975 25252961

[182] Fuster MM Esko JD. The sweet and sour of cancer: Glycans as novel therapeutic targets. Nat Rev Cancer. (2005) 5:526–42. doi: 10.1038/nrc1649 16069816

[183] Rodrigues E Macauley MS. Hypersialylation in cancer: modulation of inflammation and therapeutic opportunities. Cancers. (2018) 10:207. doi: 10.3390/cancers10060207 29912148 PMC6025361

[184] Nicoll G Avril T Lock K Furukawa K Bovin N Crocker PR. Ganglioside GD3 expression on target cells can modulate NK cell cytotoxicity via Siglec-7-dependent and -independent mechanisms. Eur J Immunol. (2003) 33:1642–8. doi: 10.1002/eji.200323930 12778482

[185] Lin CH Yeh YC Yang KD. Functions and therapeutic targets of Siglec-mediated infections, inflammations and cancers. J Formosan Med Assoc. (2021) 120:5–24. doi: 10.1016/j.jfma.2019.10.019 31882261

[186] Jiang KY Qi LL Kang FB Wang L. The intriguing roles of Siglec family members in the tumor microenvironment. biomark Res. (2022) 10:22. doi: 10.1186/s40364-022-00369-1 35418152 PMC9008986

[187] Grabenstein S Barnard KN Anim M Armoo A Weichert WS Bertozzi CR. Deacetylated sialic acids modulates immune mediated cytotoxicity via the sialic acid-Siglec pathway. Glycobiology. (2021) 31:1279–94. doi: 10.1093/glycob/cwab068 34192335

[188] Shivatare SS Shivatare VS Wong CH. Glycoconjugates: synthesis, functional studies, and therapeutic developments. Chem Rev. (2022) 122:15603–71. doi: 10.1021/acs.chemrev.1c01032 PMC9674437 36174107

[189] da Silva MAAM. Elucidating the role of glycans in leucocyte function. Universidade NOVA de Lisboa, Portugal (2015).

[190] Zhuo Ya Bellis SL. Emerging role of α2, 6-sialic acid as a negative regulator of galectin binding and function. J Biol Chem. (2011) 286:5935–41. doi: 10.1074/jbc.R110.191429 PMC3057866 21173156

[191] Cagnoni AJ Perez Saez JM Rabinovich GA Mariño KV. Turning-off signaling by siglecs, selectins, and galectins: chemical inhibition of glycan-dependent interactions in cancer. Front Oncol. (2016) 6:109. doi: 10.3389/fonc.2016.00109 27242953 PMC4865499

[192] Peixoto A Relvas-Santos M Azevedo R Santos LL Ferreira JA. Protein glycosylation and tumor microenvironment alterations driving cancer hallmarks. Front Oncol. (2019) 9:380. doi: 10.3389/fonc.2019.00380 31157165 PMC6530332

[193] Zhuo Y Chammas R Bellis SL. Sialylation of β1 integrins blocks cell adhesion to galectin-3 and protects cells against galectin-3-induced apoptosis. J Biol Chem. (2008) 283:22177–85. doi: 10.1074/jbc.M800015200 PMC2494929 18676377

[194] Olio FD MALAGoLINI N di Stefano G Minni F Marrano D Serafini-Cessi F. Increased CMP-NeuAc: Galβ1, 4GlcNAc-R α2, 6 sialyltransferase activity in human colorectal cancer tissues. Int J Cancer. (1989) 44:434–9. doi: 10.1002/ijc.2910440309 2476402

[195] Sata T Roth J Zuber C Stamm B Heitz PU. Expression of alpha 2, 6-linked sialic acid residues in neoplastic but not in normal human colonic mucosa. A lectin-gold cytochemical study with Sambucus nigra and Maackia amurensis lectins. Am J Pathol. (1991) 139:1435. 1661075 PMC1886452

[196] Recchi MA Harduin-Lepers A Boilly-Marer Y Verbert A Delannoy P. Multiplex RT-PCR method for the analysis of the expression of human sialyltransferases: application to breast cancer cells. Glycoconjugate J. (1998) 15:19–27. doi: 10.1023/A:1006983214918 9530953

[197] López-Morales D Velázquez-Márquez N Valenzuela O Santos-López G Reyes-Leyva J Vallejo-Ruiz V. Enhanced sialyltransferases transcription in cervical intraepithelial neoplasia. Investigación clínica. (2009) 50:45–53. 19418726

[198] Kaneko Y Yamamoto H Kersey DS Colley KJ Leestma JE Moskal JR. The expression of Galβ1, 4GlcNAc α2, 6 sialyltransferase and α2, 6-linked sialoglycoconjugates in human brain tumors. Acta neuropathologica. (1996) 91:284–92. doi: 10.1007/s004010050427 8834541

[199] Lise M Belluco C Perera SP Patel R Thomas P Ganguly A. Clinical correlations of α2, 6-sialyltransferase expression in colorectal cancer patients. Hybridoma. (2000) 19:281–6. doi: 10.1089/027245700429828 11001400

[200] Shah MH Telang SD Shah PM Patel PS. Tissue and serum α2-3-and α2-6-linkage specific sialylation changes in oral carcinogenesis. Glycoconjugate J. (2008) 25:279–90. doi: 10.1007/s10719-007-9086-4 18158621

[201] Shaikh FM. (2008). Role of variant sialylation in regulating tumor cell behavior: The University of Alabama at Birmingham.

[202] De Oliveira JT De Matos AJ Santos AL Pinto R Gomes J Hespanhol V. Sialylation regulates galectin-3/ligand interplay during mammary tumour progression—a case of targeted uncloaking. Int J Dev Biol. (2011) 55:823. doi: 10.1387/ijdb.113359jt 22161838

[203] Wang H-s Wang L-h. The expression and significance of Gal-3 and MUC1 in colorectal cancer and colon cancer. OncoTargets Ther. (2015), 1893–8. doi: 10.2147/OTT.S83502 PMC4524583 26251612

[204] Zheng D Hu Z He F Gao C Xu L Zou H. Downregulation of galectin-3 causes a decrease in uPAR levels and inhibits the proliferation, migration and invasion of hepatocellular carcinoma cells. Oncol Rep. (2014) 32:411–8. doi: 10.3892/or.2014.3170 24807674

[205] Hittelet A Legendre H Nagy N Bronckart Y Pector JC Salmon I. Upregulation of galectins-1 and-3 in human colon cancer and their role in regulating cell migration. Int J Cancer. (2003) 103:370–9. doi: 10.1002/ijc.v103:3 12471620

[206] Sakaki M Fukumori T Fukawa T Elsamman E Shiirevnyamba A Nakatsuji H. Clinical significance of Galectin-3 in clear cell renal cell carcinoma. J Med Invest. (2010) 57:152–7. doi: 10.2152/jmi.57.152 20299755

[207] Zaia Povegliano L Oshima CTF de Oliveira Lima F Andrade Scherholz PL Manoukian Forones N. Immunoexpression of galectin-3 in colorectal cancer and its relationship with survival. J gastrointestinal Cancer. (2011) 42:217–21. doi: 10.1007/s12029-010-9189-1 20635166

[208] Dallolio F Chiricolo M Lollini P Lau JT. Human colon cancer cell lines permanently expressing α2, 6-sialylated sugar chains by transfection with rat β-galactoside α2, 6 sialyltransferase cDNA. Biochem Biophys Res Commun. (1995) 211:554–61. doi: 10.1006/bbrc.1995.1849 7794269

[209] Büll C Stoel MA den Brok MH Adema GJ. Sialic acids sweeten a tumor’s life. Cancer Res. (2014) 74:3199–204. doi: 10.1158/0008-5472.CAN-14-0728 24830719

[210] Daly J Sarkar S Natoni A Henderson R Swan D Carlsten M. Hypersialylation protects multiple myeloma cells from NK cell-mediated immunosurveillance and this can be overcome by targeted desialylation using a sialyltransferase inhibitor. Blood. (2019) 134:138–8. doi: 10.1182/blood-2019-126613

[211] Julien S Lagadec C Krzewinski-Recchi MA Courtand G Bourhis XL Delannoy P. Stable expression of sialyl-Tn antigen in T47-D cells induces a decrease of cell adhesion and an increase of cell migration. Breast Cancer Res Treat. (2005) 90:77–84. doi: 10.1007/s10549-004-3137-3 15770530

[212] Ling AJW Chang LS Babji AS Latip J Koketsu M Lim SJ. Review of sialic acid’s biochemistry, sources, extraction and functions with special reference to edible bird’s nest. Food Chem. (2022) 367:130755. doi: 10.1016/j.foodchem.2021.130755 34390910

[213] Galvão RP Zong H. Inflammation and gliomagenesis: bi-directional communication at early and late stages of tumor progression. Curr pathobiol Rep. (2013) 1:19–28. doi: 10.1007/s40139-012-0006-3 23538742 PMC3607461

[214] Ghosh M Hazarika P Dhanya SJ Pooja D Kulhari H. Exploration of sialic acid receptors as a potential target for cancer treatment: A comprehensive review. Int J Biol Macromolecules. (2023), 128415. doi: 10.1016/j.ijbiomac.2023.128415 38029891

[215] Ghosh M Hazarika P Dhanya SJ Pooja D Kulhari H. Exploration of sialic acid receptors as a potential target for cancer treatment: A comprehensive review. Int J Biol Macromolecules. (2024), 128415. doi: 10.1016/j.ijbiomac.2023.128415 38029891

[216] Yang H Lu L Chen X. An overview and future prospects of sialic acids. Biotechnol Adv. (2021) 46:107678. doi: 10.1016/j.biotechadv.2020.107678 33285252

[217] Barnes JM Kaushik S Bainer RO Sa JK Woods EC Kai F. A tension-mediated glycocalyx-integrin feedback loop promotes mesenchymal-like glioblastoma. Nat Cell Biol. (2018) 20:1203–14. doi: 10.1038/s41556-018-0183-3 PMC6932748 30202050

[218] Locker GY Hamilton S Harris J Jessup JM Kemeny N Macdonald JS. ASCO 2006 update of recommendations for the use of tumor markers in gastrointestinal cancer. J Clin Oncol. (2006) 24:5313–27. doi: 10.1200/JCO.2006.08.2644 17060676

[219] Tanaka F Otake Y Nakagawa T Kawano Y Miyahara R Li M. Prognostic significance of polysialic acid expression in resected non-small cell lung cancer. Cancer Res. (2001) 61:1666–70. 11245481

[220] Potapenko IO Haakensen VD Lüders T Helland Å Bukholm I Sørlie T. Glycan gene expression signatures in normal and Malignant breast tissue; possible role in diagnosis and progression. Mol Oncol. (2010) 4:98–118. doi: 10.1016/j.molonc.2009.12.001 20060370 PMC5527897

[221] Hutchinson WL Du MQ Johnson PJ Williams R. Fucosyltransferases: differential plasma and tissue alterations in hepatocellular carcinoma and cirrhosis. Hepatology. (1991) 13:683–8. doi: 10.1002/hep.1840130412 1849114

[222] Rezaei M Martins Cavaco AC Stehling M Nottebaum A Brockhaus K Caliandro MF. Extracellular vesicle transfer from endothelial cells drives VE-Cadherin expression in breast cancer cells, thereby causing heterotypic cell contacts. Cancers. (2020) 12:2138. doi: 10.3390/cancers12082138 32752204 PMC7463713

[223] Paszek MJ DuFort CC Rossier O Bainer R Mouw JK Godula K. The cancer glycocalyx mechanically primes integrin-mediated growth and survival. Nature. (2014) 511:319–25. doi: 10.1038/nature13535 PMC4487551 25030168

[224] Tinder TL Subramani DB Basu GD Bradley JM Schettini J Million A. MUC1 enhances tumor progression and contributes toward immunosuppression in a mouse model of spontaneous pancreatic adenocarcinoma. J Immunol. (2008) 181:3116–25. doi: 10.4049/jimmunol.181.5.3116 PMC2625292 18713982

[225] Coussens LM Werb Z. Inflammation and cancer. Nature. (2002) 420:860–7. doi: 10.1038/nature01322 PMC2803035 12490959

[226] Wright RD Cooper D. Glycobiology of leukocyte trafficking in inflammation. Glycobiology. (2014) 24:1242–51. doi: 10.1093/glycob/cwu101 25258391

[227] Cummings RD Liu FT Vasta GR Varki A Esko JD Stanley P. Essentials of glycobiology. United States: Cold spring harbor Press (2009), pp. 633–48. 20301239

[228] Butler PJ Bhatnagar A. Mechanobiology of the abluminal glycocalyx. Biorheology. (2019) 56:101–12. doi: 10.3233/BIR-190212 31561318

[229] Dennis JW Laferté S Waghorne C Breitman ML Kerbel RS. [amp]]beta;1-6 branching of Asn-linked oligosaccharides is directly associated with metastasis. Science. (1987) 236:582–5. doi: 10.1126/science.2953071 2953071

[230] Dusoswa SA Verhoeff J Abels E Méndez-Huergo SP Croci DO Kuijper LH. Glioblastomas exploit truncated O-linked glycans for local and distant immune modulation via the macrophage galactose-type lectin. Proc Natl Acad Sci. (2020) 117:3693–703. doi: 10.1073/pnas.1907921117 PMC7035608 32019882

[231] Demetriou M Nabi IR Coppolino M Dedhar S Dennis JW. Reduced contact-inhibition and substratum adhesion in epithelial cells expressing GlcNAc-transferase V. J Cell Biol. (1995) 130:383–92. doi: 10.1083/jcb.130.2.383 PMC2199932 7615638

[232] Hirabayashi Y. A world of sphingolipids and glycolipids in the brain–novel functions of simple lipids modified with glucose. Proc Jpn Acad Ser B Phys Biol Sci. (2012) 88:129–43. doi: 10.2183/pjab.88.129 PMC3406307 22498977

[233] Greco B Malacarne V De Girardi F Scotti GM Manfredi F Angelino E. Disrupting N-glycan expression on tumor cells boosts chimeric antigen receptor T cell efficacy against solid Malignancies. Sci Trans Med. (2022) 14:eabg3072. doi: 10.1126/scitranslmed.abg3072 35044789

[234] Heitger A Ladisch S. Gangliosides block antigen presentation by human monocytes. Biochim Biophys Acta (BBA)-Lipids Lipid Metab. (1996) 1303:161–8. doi: 10.1016/0005-2760(96)00091-4 8856046

[235] Kimata H Yoshida A. Differential effects of gangliosides on Ig production and proliferation by human B cells. Blood (1994) 84(4):1193–200. doi: 10.1182/blood.V84.4.1193 8049434

[236] Shurer CR Colville MJ Gupta VK Head SE Kai F Lakins JN. Genetically encoded toolbox for glycocalyx engineering: tunable control of cell adhesion, survival, and cancer cell behaviors. ACS Biomater Sci Eng. (2018) 4:388–99. doi: 10.1021/acsbiomaterials.7b00037 PMC5966047 29805991

[237] Hakomori S. Glycosylation defining cancer Malignancy: new wine in an old bottle. Proc Natl Acad Sci. (2002) 99:10231–3. doi: 10.1073/pnas.172380699 PMC124893 12149519

[238] Takamatsu S. Unusually high expression of N-acetylglucosaminyltransferase- IVa in human choriocarcinoma cell lines: A possible enzymatic basis of the formation of abnormal biantennary sugar chain. Cancer Res. (1999) 59:3949–53. 10463590

[239] Marhuenda E. Glioma stem cells invasive phenotype at optimal stiffness is driven by MGAT5 dependent mechanosensing. J Exp Clin Cancer Res. (2021) 40:139. doi: 10.1186/s13046-021-01925-7 33894774 PMC8067292

[240] Granovsky M. Suppression of tumor growth and metastasis in Mgat5-deficient mice. Nat Med. (2000) 6:306–12. doi: 10.1038/73163 10700233

[241] Zhao Y. Branched N-glycans regulate the biological functions of integrins and cadherins. FEBS J. (2008) 275:1939–48. doi: 10.1111/j.1742-4658.2008.06346.x 18384383

[242] de-Souza-Ferreira M Ferreira ÉE de-Freitas-Junior JCM. Aberrant N-glycosylation in cancer: MGAT5 and β1, 6-GlcNAc branched N-glycans as critical regulators of tumor development and progression. Cell Oncol. (2023) 46:481–501. doi: 10.1007/s13402-023-00770-4 PMC12974675 36689079

[243] Li Y Liu Y Zhu H Chen X Tian M Wei Y. N-acetylglucosaminyltransferase I promotes glioma cell proliferation and migration through increasing the stability of the glucose transporter GLUT1. FEBS Lett. (2020) 594:358–66. doi: 10.1002/1873-3468.13596 31494931

[244] Xiang T Qiao M Xie J Li Z Xie H. Emerging roles of the unique molecular chaperone Cosmc in the regulation of health and disease. Biomolecules. (2022) 12:1732. doi: 10.3390/biom12121732 36551160 PMC9775496

[245] Marciel MP Haldar B Hwang J Bhalerao N Bellis SL. Role of tumor cell sialylation in pancreatic cancer progression. Adv Cancer Res. (2023) 157:123–55. doi: 10.1016/bs.acr.2022.07.003 PMC11342334 36725107

[246] Sun YF Zhang LC Niu RZ Chen L Xia QJ Xiong LL. Predictive potentials of glycosylation-related genes in glioma prognosis and their correlation with immune infiltration. Sci Rep. (2024) 14:4478. doi: 10.1038/s41598-024-51973-0 38396140 PMC10891078

[247] Gao T Du T Hu X Dong X Li L Wang Y. Cosmc overexpression enhances Malignancies in human colon cancer. J Cell Mol Med. (2020) 24:362–70. doi: 10.1111/jcmm.14740 PMC6933370 31633299

[248] Lee JJ Chen CH Chen YH Huang MJ Huang J Hung JS. COSMC is overexpressed in proliferating infantile hemangioma and enhances endothelial cell growth via VEGFR2. PloS One. (2013) 8:e56211. doi: 10.1371/journal.pone.0056211 23424651 PMC3570459

[249] Schömel N Gruber L Alexopoulos SJ Trautmann S Olzomer EM Byrne FL. UGCG overexpression leads to increased glycolysis and increased oxidative phosphorylation of breast cancer cells. Sci Rep. (2020) 10:8182. doi: 10.1038/s41598-020-65182-y 32424263 PMC7234995

[250] D'Angelo G Capasso S Sticco L Russo D. Glycosphingolipids: synthesis and functions. FEBS J. (2013) 280:6338–53. doi: 10.1111/febs.12559 24165035

[251] Liang Y-J Yang B-C Chen J-M Lin Y-H Huang C-L Cheng Y-Y. Changes in glycosphingolipid composition during differentiation of human embryonic stem cells to ectodermal or endodermal lineages. Stem Cells. (2011) 29:1995–2004. doi: 10.1002/stem.750 21956927

[252] Yeh S-C Wang P-Y Lou Y-W Khoo K-H Hsiao M Hsu T-L. Glycolipid GD3 and GD3 synthase are key drivers for glioblastoma stem cells and tumorigenicity. Proc Natl Acad Sci USA. (2016) 113:5592–7. doi: 10.1073/pnas.1604721113 PMC4878508 27143722

[253] Birks SM Danquah JO King L Vlasak R Gorecki DC Pilkington GJ. Targeting the GD3 acetylation pathway selectively induces apoptosis in glioblastoma. Neuro-oncology. (2011) 13:950–60. doi: 10.1093/neuonc/nor108 PMC3158019 21807667

[254] Fleurence J Cochonneau D Fougeray S Oliver L Geraldo F Terme M. Targeting and killing glioblastoma with monoclonal antibody to O-acetyl GD2 ganglioside. Oncotarget. (2016) 7:41172–85. doi: 10.18632/oncotarget.9226 PMC5173050 27172791

[255] Kudelka MR Ju T Heimburg-Molinaro J Cummings RD. Simple sugars to complex disease-mucin-type O-glycans in cancer. Adv Cancer Res. (2015) 126:53–135. doi: 10.1016/bs.acr.2014.11.002 25727146 PMC5812724

[256] Zhang L Underhill CB Chen L. Hyaluronan on the surface of tumor cells is correlated with metastatic behavior. Cancer Res. (1995) 55:428–33. 7529138

[257] Fieber C Baumann P Vallon R Termeer C Simon JC Hofmann M. Hyaluronan-oligosaccharide-induced transcription of metalloproteases. J Cell Sci. (2004) 117:359–67. doi: 10.1242/jcs.00831 14657275

[258] Anttila MA Tammi RH Tammi MI Syrjänen KJ Saarikoski SV Kosma VM. High levels of stromal hyaluronan predict poor disease outcome in epithelial ovarian cancer. Cancer Res. (2000) 60:150–5. 10646867

[259] Lopez JI Camenisch TD Stevens MV Sands BJ McDonald J Schroeder JA. CD44 attenuates metastatic invasion during breast cancer progression. Cancer Res. (2005) 65:6755–63. doi: 10.1158/0008-5472.CAN-05-0863 16061657

[260] Naor D Wallach-Dayan SB Zahalka MA Sionov RV. Involvement of CD44, a molecule with a thousand faces, in cancer dissemination. Semin Cancer Biol. (2008) 18:260–7. doi: 10.1016/j.semcancer.2008.03.015 18467123

[261] Barnes JM Przybyla L Weaver VM. Tissue mechanics regulate brain development, homeostasis and disease. J Cell Sci. (2017) 130:71–82. doi: 10.1242/jcs.191742 28043968 PMC5394781

[262] Buffone A Mondal N Gupta R McHugh KP Lau JT Neelamegham S. Silencing alpha1,3-fucosyltransferases in human leukocytes reveals a role for FUT9 enzyme during E-selectin-mediated cell adhesion. J Biol Chem. (2013) 288:1620–33. doi: 10.1074/jbc.M112.400929 PMC3548472 23192350

[263] Sander JD Keith Joung J. CRISPR-Cas systems for editing, regulating and targeting genomes. Nat Biotechnol. (2014) 32:347–55. doi: 10.1038/nbt.2842 PMC4022601 24584096

[264] Vester-Christensen MB Halim A Joshi HJ Steentoft C Bennett EP Levery SB. Mining the O-mannose glycoproteome reveals cadherins as major O-mannosylated glycoproteins. Proc Natl Acad Sci U.S.A. (2013) 110:21018–23. doi: 10.1073/pnas.1313446110 PMC3876253 24101494

[265] Steentoft C Bennett EP Schjoldager KT Vakhrushev SY Wandall HH Clausen H. Precision genome editing: a small revolution for glycobiology. Glycobiology. (2014) 24:663–80. doi: 10.1093/glycob/cwu046 24861053

[266] Kuan SF Byrd JC Basbaum C Kim YS. Inhibition of mucin glycosylation by aryl-N-acetyl-α-galactosaminides in human colon cancer cells. J Biol Chem. (1989) 264:19271–7. doi: 10.1016/S0021-9258(19)47297-9 2509474

[267] Qazi H Shi Z-D Song JW Cancel LM Huang P Zeng Ye. Heparan sulfate proteoglycans mediate renal carcinoma metastasis. Int J Cancer. (2016) 139:2791–801. doi: 10.1002/ijc.v139.12 PMC7718768 27543953

[268] Wang X LH Li J Su Y Xu L. Muc1 promotes migration and lung metastasis of melanoma cells. Am J Cancer Res. (2015) 5:2590–604. PMC4633892 26609470

[269] Moran H Cancel LM Mayer MA Qazi H Munn LL Tarbell JM. The cancer cell glycocalyx proteoglycan Glypican-1 mediates interstitial flow mechanotransduction to enhance cell migration and metastasis. Biorheology. (2019) 56(2-3):151–61. doi: 10.3233/bir-180203 31256115

[270] Elkashef SM Allison SJ Sadiq M Basheer HA Ribeiro Morais G Loadman PM. Polysialic acid sustains cancer cell survival and migratory capacity in a hypoxic environment. Sci Rep. (2016), 33026. doi: 10.1038/srep33026 27611649 PMC5017143

[271] Paszek MJ Boettiger D Weaver VM Hammer DA. Integrin clustering is driven by mechanical resistance from the glycocalyx and the substrate. PloS Comput Biol. (2009) 5:e1000604. doi: 10.1371/journal.pcbi.1000604 20011123 PMC2782178

[272] Xu GK Qian J Hu J. The glycocalyx promotes cooperative binding and clustering of adhesion receptors. Soft Matter. (2016) 12:4572–83. doi: 10.1039/C5SM03139G 27102288

[273] Sökeland G Schumacher U. The functional role of integrins during intraand extravasation within the metastatic cascade. Mol Cancer. (2019) 18:1–19. 30657059 10.1186/s12943-018-0937-3 PMC6337777

[274] Chen MB Lamar JM Li R Hynes RO Kamm RD. Elucidation of the roles of tumor integrin β1 in the extravasation stage of the metastasis cascade. Cancer Res. (2016) 76:2513–24. doi: 10.1158/0008-5472.CAN-15-1325 PMC4873393 26988988

[275] Van Sluis GL Nieuwdorp M Kamphuisen PW van der Vlag J Van Noorden CJ Spek CA. A low molecular weight heparin inhibits experimental metastasis in mice independently of the endothelial glycocalyx. PloS One. (2010) 5:e11200. doi: 10.1371/journal.pone.0011200 20574516 PMC2888573

[276] Freeman SA GJ Furuya W Woods EC Bertozzi CR Bergmeier W Hinz B. Integrins form an expanding diffusional barrier that coordinates phagocytosis. Cell. (2022) 164:128–40. doi: 10.1016/j.cell.2015.11.048 PMC4715264 26771488

[277] Spicer AP RG Lidner TK Gendler SJ. Delayed mammary tumor progression in Muc-1 null mice. J Biol Chem. (1995) 270:30093–101. doi: 10.1074/jbc.270.50.30093 8530414

[278] Mitra SK Hanson DA Schlaepfer DD. Focal adhesion kinase: in command and control of cell motility. Nat Rev Mol Cell Biol. (2005) 6:56–68. doi: 10.1038/nrm1549 15688067

[279] DuFort CC Paszek MJ Weaver VM. Balancing forces: architectural control of mechanotransduction. Nat Rev Mol Cell Biol. (2011) 12:308–19. doi: 10.1038/nrm3112 PMC3564968 21508987

[280] Moreno-Layseca P Streuli CH. Signalling pathways linking integrins with cell cycle progression. Matrix Biol. (2014) 34:144–53. doi: 10.1016/j.matbio.2013.10.011 24184828

[281] Valiente M Ahluwalia MS Boire A Brastianos PK Goldberg SB Lee EQ. The evolving landscape of brain metastasis. Trends Cancer. (2018) 4:176–96. doi: 10.1016/j.trecan.2018.01.003 PMC6602095 29506669

[282] Boire A Brastianos PK Garzia L Valiente M. Brain metastasis. Nat Rev Cancer. (2020) 20:4–11. doi: 10.1038/s41568-019-0220-y 31780784

[283] Lah TT Novak M Breznik B. Brain Malignancies: Glioblastoma and brain metastases. Semin Cancer Biol. (2020) 60:262–73. doi: 10.1016/j.semcancer.2019.10.010 31654711

[284] Walker MR Goel HL Mukhopadhyay D Chhoy P Karner ER Clark JL. O-linked α2, 3 sialylation defines stem cell populations in breast cancer. Sci Adv. (2022) 8:eabj9513. 34995107 10.1126/sciadv.abj9513 PMC8741191

[285] Isaji T Gu J. Novel regulatory mechanisms of N-glycan sialylation: Implication of integrin and focal adhesion kinase in the regulation. Biochim Biophys Acta (BBA)-General Subj. (2024) 1868:130617. doi: 10.1016/j.bbagen.2024.130617 38614280

[286] Schildhauer P Selke P Staege MS Harder A Scheller C Strauss C. Glycation interferes with the expression of sialyltransferases and leads to increased polysialylation in glioblastoma cells. Cells. (2023) 12:2758. doi: 10.3390/cells12232758 38067186 PMC10706364

[287] Rosa-Fernandes L Oba-Shinjo SM Macedo-da-Silva J Marie SKN Palmisano G. Aberrant protein glycosylation in brain cancers, with emphasis on glioblastoma. In: Understanding PTMs in neurodegenerative diseases. Springer International Publishing, Cham (2022). p. 39–70. 10.1007/978-3-031-05460-0_4 36029403

[288] Kasprowicz A Sophie GD Lagadec C Delannoy P. Role of GD3 synthase ST8Sia I in cancers. Cancers. (2022) 14:1299. doi: 10.3390/cancers14051299 35267607 PMC8909605

[289] Liu J Zheng X Pang X Li L Wang J Yang C. Ganglioside GD3 synthase (GD3S), a novel cancer drug target. Acta Pharm Sin B. (2018) 8:713–20. doi: 10.1016/j.apsb.2018.07.009 PMC6147802 30245960

[290] Paszek MJ Zahir N Johnson KR Lakins JN Rozenberg GI Gefen A. Tensional homeostasis and the Malignant phenotype. Cancer Cell. (2005) 8:241–54. doi: 10.1016/j.ccr.2005.08.010 16169468

[291] Katsumi A Orr AW Tzima E Schwartz MA. Integrins in mechanotransduction. J Biol Chem. (2004) 279:12001–4. doi: 10.1074/jbc.R300038200 14960578

[292] Tzima E Del Pozo MA Shattil SJ Chien S Schwartz MA. Activation of integrins in endothelial cells by fluid shear stress mediates Rho-dependent cytoskeletal alignment. EMBO J. (2001) 20:4639–47. doi: 10.1093/emboj/20.17.4639 PMC125600 11532928

[293] Mekhdjian AH Kai F Rubashkin MG Prahl LS Przybyla LM McGregor AL. Integrin-mediated traction force enhances paxillin molecular associations and adhesion dynamics that increase the invasiveness of tumor cells into a three-dimensional extracellular matrix. Mol Biol Cell. (2017) 28:1467–88. doi: 10.1091/mbc.e16-09-0654 PMC5449147 28381423

[294] Laklai H Miroshnikova YA Pickup MW Collisson EA Kim GE Barrett AS. Genotype tunes pancreatic ductal adenocarcinoma tissue tension to induce matricellular fibrosis and tumor progression. Nat Med. (2016) 22:497–505. doi: 10.1038/nm.4082 27089513 PMC4860133

[295] Miroshnikova YA Mouw JK Barnes JM Pickup MW Lakins JN Kim Y. Tissue mechanics promote IDH1-dependent HIF1alpha-tenascin C feedback to regulate glioblastoma aggression. Nat Cell Biol. (2016) 18:1336–45. doi: 10.1038/ncb3429 PMC5361403 27820599

[296] Tian B Luo Q Ju Y Song G. A soft matrix enhances the cancer stem cell phenotype of HCC cells. Int J Mol Sci. (2019) 20:2831. doi: 10.3390/ijms20112831 31185668 PMC6600428

[297] Kim Y Kumar S. CD44-mediated adhesion to hyaluronic acid contributes to mechanosensing and invasive motility. Mol Cancer Res. (2014) 12:1416–29. doi: 10.1158/1541-7786.MCR-13-0629 PMC4201971 24962319

[298] Zhang L Huang G Li X Zhang Y Jiang Y Shen J. Hypoxia induces epithelial-mesenchymal transition via activation of SNAI1 by hypoxia-inducible factor-1α in hepatocellular carcinoma. BMC Cancer. (2013) 13:1–9. doi: 10.1186/1471-2407-13-108 23496980 PMC3614870

[299] Chen S Chen JZ Zhang JQ Chen HX Yan ML Huang L. Hypoxia induces TWIST-activated epithelial-mesenchymal transition and proliferation of pancreatic cancer cells *in vitro* and in nude mice. Cancer Lett. (2016) 383:73–84. doi: 10.1016/j.canlet.2016.09.027 27693633

[300] Koser DE Thompson AJ Foster SK Dwivedy A Pillai EK Sheridan GK. Mechanosensing is critical for axon growth in the developing brain. Nat Neurosci. (2016) 19:1592–8. doi: 10.1038/nn.4394 PMC5531257 27643431

[301] Doetsch F Petreanu L Caille I Garcia-Verdugo J-M Alvarez-Buylla A. EGF converts transit-amplifying neurogenic precursors in the adult brain into multipotent stem cells. Neuron. (2002) 36:1021–34. doi: 10.1016/S0896-6273(02)01133-9 12495619

[302] Shurer CR Kuo JC Roberts LM Gandhi JG Colville MJ Enoki TA. Physical principles of membrane shape regulation by the glycocalyx. Cell. (2019) 177:1757–1770 e21. doi: 10.1016/j.cell.2019.04.017 31056282 PMC6768631

[303] Cancer Genome Atlas Research Network. Comprehensive, integrative genomic analysis of diffuse lower-grade gliomas. New Engl J Med. (2015) 372:2481–98. doi: 10.1056/NEJMoa1402121 PMC4530011 26061751

[304] Barkovskaya A Buffone A Žídek M Weaver VM. Proteoglycans as mediators of cancer tissue mechanics. Front Cell Dev Biol. (2020) 8. doi: 10.3389/fcell.2020.569377 PMC7734320 33330449

[305] Karamanos NK Theocharis AD Piperigkou Z Manou D Passi A Skandalis SS. A guide to the composition and functions of the extracellular matrix. FEBS J. (2021) 288:6850–912. doi: 10.1111/febs.v288.24 33605520

[306] Harisi R Jeney A. Extracellular matrix as target for antitumor therapy. OncoTargets Ther. (2015), 1387–98. doi: 10.2147/OTT.S48883 PMC4467640 26089687

[307] Kariya Y Kariya Y Gu J. Roles of integrin α6β4 glycosylation in cancer. Cancers. (2017) 9:79. doi: 10.3390/cancers9070079 28678156 PMC5532615

[308] Seales EC Jurado GA Brunson BA Wakefield JK Frost AR Bellis SL. Hypersialylation of β1 integrins, observed in colon adenocarcinoma, may contribute to cancer progression by up-regulating cell motility. Cancer Res. (2005) 65:4645–52. doi: 10.1158/0008-5472.CAN-04-3117 15930282

[309] Yousefi H Vatanmakanian M Mahdiannasser M Mashouri L Alahari NV Monjezi MR. Understanding the role of integrins in breast cancer invasion, metastasis, angiogenesis, and drug resistance. Oncogene. (2021) 40:1043–63. doi: 10.1038/s41388-020-01588-2 33420366

[310] Hill CAS. Interactions between endothelial selectins and cancer cells regulate metastasis. Front bioscience. (2011) 16:3233–51. doi: 10.2741/3909 21622232

[311] Ales E Sackstein R. The biology of E-selectin ligands in leukemogenesis. Adv Cancer Res. (2023) 157:229–50. doi: 10.1016/bs.acr.2022.07.001 PMC12795549 36725110

[312] Lowe JB. Glycan-dependent leukocyte adhesion and recruitment in inflammation. Curr Opin Cell Biol. (2003) 15:531–8. doi: 10.1016/j.ceb.2003.08.002 14519387

[313] Bhide GP Colley KJ. Sialylation of N-glycans: mechanism, cellular compartmentalization and function. Histochem Cell Biol. (2017) 147:149–74. doi: 10.1007/s00418-016-1520-x PMC7088086 27975143

[314] Mondal N Buffone A Stolfa G Antonopoulos A Lau JTY Haslam SM. ST3Gal-4 is the primary sialyltransferase regulating the synthesis of E-, P-, and L-selectin ligands on human myeloid leukocytes. Blood. (2014) 125:687–96. doi: 10.1182/blood-2014-07-588590 PMC4304113 25498912

[315] Mondal N Stolfa G Antonopoulos A Zhu Y Wang S-S Buffone A. Glycosphingolipids on human myeloid cells stabilize E-selectin–dependent rolling in the multistep leukocyte adhesion cascade. Arteriosclerosis Thrombosis Vasc Biol. (2016) 36:718–27. doi: 10.1161/ATVBAHA.115.306748 PMC5567847 26868209

[316] Sipkins DA Wei X Wu JW Runnels JM Côté D Means TK. *In vivo* imaging of specialized bone marrow endothelial microdomains for tumour engraftment. Nature. (2005) 435:969–73. doi: 10.1038/nature03703 PMC2570168 15959517

[317] Esposito M Mondal N Greco TM Wei Y Spadazzi C Lin S-C. Bone vascular niche E-selectin induces mesenchymal–epithelial transition and Wnt activation in cancer cells to promote bone metastasis. Nat Cell Biol. (2019) 21:627–39. doi: 10.1038/s41556-019-0309-2 PMC6556210 30988423

[318] Ghosh S. Sialic acids and sialoglycans in endocrinal disorders. Sialic Acids and Sialoglycoconjugates in the Biology of Life, Health and Disease. United States: Academic Press (2020) p. 247–68.

[319] Ghosh S. Sialic acids: biomarkers in endocrinal cancers. Glycoconjugate J. (2015) 32:79–85. doi: 10.1007/s10719-015-9577-7 25777812

[320] Stanczak MA Rodrigues Mantuano N Kirchhammer N Sanin DE Jacob F Coelho R. Targeting cancer glycosylation repolarizes tumor-associated macrophages allowing effective immune checkpoint blockade. Sci Trans Med. (2022) 14. doi: 10.1126/scitranslmed.abj1270 PMC9812757 36322632

[321] Natoni A Bohara R Pandit A O’Dwyer M. Targeted approaches to inhibit sialylation of multiple myeloma in the bone marrow microenvironment. Front bioengineering Biotechnol. (2019) 7:252. doi: 10.3389/fbioe.2019.00252 PMC6787837 31637237

[322] RodrÍguez E Schetters STT van Kooyk Y. The tumour glyco-code as a novel immune checkpoint for immunotherapy. Nat Rev Immunol. (2018) 18:204–11. doi: 10.1038/nri.2018.3 29398707

[323] Hsu JM Li CW Lai YJ Hung MC. Posttranslational modifications of PDL1 and their applications in cancer therapy. Cancer Res. (2018) 78:6349–53. doi: 10.1158/0008-5472.CAN-18-1892 PMC6242346 30442814

[324] Xiao H Woods EC Vukojicic P Bertozzi CR. Precision glycocalyx editing as a strategy for cancer immunotherapy. Proc Natl Acad Sci United States America. (2016) 113:10304–9. doi: 10.1073/pnas.1608069113 PMC5027407 27551071

[325] Peng L. A novel immunomodulatory strategy of targeting glyco-immune checkpoints with EAGLE technology. Eur J Cancer. (2018) 03:e77. doi: 10.1158/2326-6074.TUMIMM18-PR18

[326] Stolfa G Mondal N Zhu Y Yu X Buffone A Jr Neelamegham S. Using CRISPR-Cas9 to quantify the contributions of O-glycans, N-glycans and Glycosphingolipids to human leukocyte-endothelium adhesion. Sci Rep. (2016) 6:30392. doi: 10.1038/srep30392 27458028 PMC4960646

